# The atypical cyclin-like protein Spy1 overrides p53-mediated tumour suppression and promotes susceptibility to breast tumourigenesis

**DOI:** 10.1186/s13058-019-1211-3

**Published:** 2019-12-11

**Authors:** Bre-Anne Fifield, Ingrid Qemo, Evie Kirou, Robert D. Cardiff, Lisa Ann Porter

**Affiliations:** 1 Department of Biological Sciences, University of Windsor, Windsor, ON N9B 3P4 Canada; 2 Center of Comparative Medicine, University of California, Davis, CA USA

**Keywords:** Cdk, Cyclin, Cell cycle, Tumour suppressor, Mammary gland, DNA damage

## Abstract

**Background:**

Breast cancer is the most common cancer to affect women and one of the leading causes of cancer-related deaths. Proper regulation of cell cycle checkpoints plays a critical role in preventing the accumulation of deleterious mutations. Perturbations in the expression or activity of mediators of cell cycle progression or checkpoint activation represent important events that may increase susceptibility to the onset of carcinogenesis. The atypical cyclin-like protein Spy1 was isolated in a screen for novel genes that could bypass the DNA damage response. Clinical data demonstrates that protein levels of Spy1 are significantly elevated in ductal and lobular carcinoma of the breast. We hypothesized that elevated Spy1 would override protective cell cycle checkpoints and support the onset of mammary tumourigenesis.

**Methods:**

We generated a transgenic mouse model driving expression of Spy1 in the mammary epithelium. Mammary development, growth characteristics and susceptibility to tumourigenesis were studied. In vitro studies were conducted to investigate the relationship between Spy1 and p53.

**Results:**

We found that in the presence of wild-type p53, Spy1 protein is held ‘in check’ via protein degradation, representing a novel endogenous mechanism to ensure protected checkpoint control. Regulation of Spy1 by p53 is at the protein level and is mediated in part by Nedd4. Mutation or abrogation of p53 is sufficient to allow for accumulation of Spy1 levels resulting in mammary hyperplasia. Sustained elevation of Spy1 results in elevated proliferation of the mammary gland and susceptibility to tumourigenesis.

**Conclusions:**

This mouse model demonstrates for the first time that degradation of the cyclin-like protein Spy1 is an essential component of p53-mediated tumour suppression. Targeting cyclin-like protein activity may therefore represent a mechanism of re-sensitizing cells to important cell cycle checkpoints in a therapeutic setting.

## Introduction

Breast cancer is the most prevalent form of cancer to affect women and represents the second leading cause of cancer-related mortality among this population. Increased incidence of breast cancer in women can be attributed to the complex cellular changes the female mammary gland undergoes throughout life in response to hormonal cues. A delicate balance of cell cycle progression and inhibition is required at each of these periods of development to ensure maintenance of genomic stability, a crucial factor in the inhibition of tumourigenesis. Women with inherited mutations in genes that play fundamental roles in recognition of DNA damage and activation of DNA repair pathways have an elevated risk of breast cancer. Hence, understanding how mammary epithelial cells monitor and respond to changes in genomic instability throughout development may reveal novel factors that predispose women to carcinogenesis.

The tumour suppressor p53 plays a critical role in DNA repair mechanisms, functioning to initiate arrest, repair and apoptotic programmes [1–4]. Over 50% of human cancers contain a mutation in the *TP53* gene; individuals with Li-Fraumeni syndrome harbouring germline mutations in *TP53* are at an increased risk of developing cancer, including breast cancer, and mouse models with germline knockout of p53 develop normally; however, spontaneous tumours occur at an increased rate [5–10]. Thus, the inability of a cell to efficiently recognize and repair DNA damage plays a key role in the onset of tumourigenesis. Although p53 is widely mutated in human cancers and individuals with Li-Fraumeni syndrome have an elevated risk of breast cancer, this population comprises a small percentage of those with breast cancer, stressing the importance for cooperating genes in the initiation and/or progression of disease [11]. It is likely that these genes also play critical roles in normal cellular events that regulate proliferation, checkpoint activation and detection and repair of DNA damage, as aberrant expression of such genes would lead to genomic instability. Thus, it is of high importance to identify additional genes that may be implicated in breast cancer susceptibility.

An atypical cyclin-like protein Spy1 (also called Ringo, Speedy1; gene SPDYA) was initially discovered in a screen for genes that would override cell death following ultraviolet (UV) radiation in a rad1-deficient strain of *S. pombe*, suggesting a role for this protein in overriding critical checkpoint responses following DNA damage [12]. Several groups have demonstrated that Spy1 is capable of inhibiting apoptosis and promoting progression through both G1/S and G2/M phase of the cell cycle [13–16]. Spy1 function is currently attributed to the direct binding to the cyclin-dependent kinases (Cdks), activating both Cdk1 and Cdk2 independent of threonine 161/160 phosphorylation status [14–19]. In the mammary gland, Spy1 protein levels are tightly regulated through development, being high during proliferative stages and downregulated at the onset of differentiation [20]. Interestingly, levels rise at the onset of involution, a period of development characterized by apoptosis and the triggering of regenerative processes [20]. When overexpressed in immortalized cells with a mutated p53 and transplanted in cleared fat pad assays, elevated levels of Spy1 protein lead to precocious development of the mammary gland, disrupt normal morphogenesis and accelerate mammary tumourigenesis [20]. Spy1 is elevated in human breast cancer [21, 22], as well as several other forms of cancer including the brain, liver and blood [23–25]. The ability of Spy1 to both enhance proliferation and override apoptosis and critical checkpoint responses provides further support for this finding. Spy1 may serve as an important mediator of the DNA damage response (DDR) in maintaining the proper balance of cellular proliferation; thus, deregulation of Spy1 may play a crucial role in the transition from precancerous to cancerous cell.

In this work, we drive Spy1 overexpression in the mammary gland using the mouse mammary tumour virus (MMTV) promoter (MMTV-Spy1). We find that while glands are significantly more proliferative, there is no gross overall defect or pathology to the gland. Importantly, when hit with chemical carcinogens, MMTV-Spy1 mice accumulate more DNA damage and have elevated susceptibility to mammary tumour formation. We noted that in this model endogenous wild-type-p53 was capable of keeping levels of Spy1 protein in check. We proceed to demonstrate a novel negative feedback loop with p53. This work demonstrates that tight regulation over the levels of cyclin-like proteins is a critical component of mammary tumour suppression and loss of control promotes hyperplastic growth and tumour initiation in the breast.

## Materials and methods

### Construction of transgene

The MMTV-Spy1 transgene was prepared as follows. Site-directed mutagenesis was utilized to create an additional EcoRI site in Flag-Spy1A-pLXSN [26] to allow for subsequent removal of the Spy1 coding sequence using EcoRI digestion. The MMTV-SV40-TRPS-1 vector (kind gift from Dr. Gabriel E DiMattia) was digested with EcoRI to remove the TRPS-1 coding sequence to allow for subsequent ligation of the Spy1 coding sequence into the MMTV-SV40 backbone.

### Generation and maintenance of MMTV-Spy1 transgenic mice

MMTV-Spy1 (B6CBAF1/J-Tg (MMTV-Spy1)1Lport319, B6CBAF1/J-Tg (MMTV-Spy1)1Lport410 and B6CBAF1/J-Tg (MMTV-Spy1)1Lport413) mice were generated as follows: the MMTV-Spy1 vector was digested with XhoI and SpeI to isolate the MMTV-Spy1 transgene fragment and remove the remainder of the vector backbone. The transgene was sent to the London Regional Transgenic and Gene Targeting Facility for pronuclear injections in B6CBAF1/J hybrid embryos. Identification of founders and subsequent identification of positive pups was performed by PCR analysis. PCR was performed by adding 50 ng of genomic tail DNA to a 25 μL reaction (1× PCR buffer, 2 mM MgSO4, 0.2 mM dNTP, 0.04 U/μL BioBasic Taq Polymerase, 0.4 μM forward primer [5′CCCAAGGCTTAAGTAAGTTTTTGG 3′], 0.4 μM reverse primer [5′ GGGCATAAGCACAGATAAAACACT 3′], 1% DMSO) (NCI Mouse Repository). Cycling conditions were as follows: 94 °C for 3 min, 40 cycles of 94 °C for 1 min, 55 °C for 2 min and 72 °C for 1 min, and a final extension of 72 °C for 3 min. Mice were maintained hemizygously following the Canadian Council on Animal Care Guidelines under animal utilization protocol 14-22 approved by the University of Windsor.

### Primary cell harvest and culture

Mammary tissue of the inguinal mammary gland was dissected, and primary mammary epithelial cells were isolated as described [27]. Cells were also seeded on attachment plates in media containing 5% foetal bovine serum, 5 ng/mL EGF, 5 μg/mL insulin, 50 μg/mL gentamycin and 1% penicillin/streptomycin (P/S) in DMEM-F12 for BrdU incorporation assays conducted 1 week after isolation of the cells.

### Mammary fat pad transplantation

The p53 knockout mouse, B6.129S2-Trp53tm1Tyj/J, was purchased from Jackson Laboratory (002101) [10]. Mammary epithelial cells were isolated from 8-week-old mice and transplanted into the cleared glands of 3-week-old B6CBAF1/J females. Successful clearing was monitored via the addition of a cleared gland with no injected cells.

### Cell culture

Human embryonic kidney cells, HEK-293 (CRL-1573; ATCC), and MDA-MB-231 (HTB-26; ATCC) and MCF7 (HTB-22; ATCC) were cultured in Dulbecco’s modified Eagle’s medium (DMEM; D5796; Sigma Aldrich) supplemented with 10% foetal bovine serum (FBS; F1051; Sigma Aldrich) and 10% calf serum (C8056; Sigma Aldrich), respectively, and 1% P/S. Mouse mammary epithelial cells, HC11 (provided by Dr. C. Shemanko), were maintained in RPMI supplemented with 10% newborn calf serum, 5 μg/mL insulin, 10 ng/mL EGF and 1% penicillin/streptomycin. All cell lines were maintained at 5% CO_2_ at 37 °C. A BioRad TC10 Automated Cell Counter was used to assess cell viability via trypan blue exclusion. MG132 (Sigma Aldrich) was used at a concentration of 10 μM and was added 12–16 h post transfection. Cell lines purchased from ATCC were authenticated via ATCC. Cells were subject to routine mycoplasma testing. All cell lines were used within three passages of thawing.

### Plasmids

The pEIZ plasmid was a kind gift from Dr. B. Welm, and the pEIZ-Flag-Spy1 vector was generated as previously described [24]. pCS3 and Myc-Spy1-pCS3 plasmids were generated as previously described [14], the Myc-Spy1-TST pCS3 plasmid was generated as previously described [28] and the p53-GFP backbone was purchased from Addgene (11770) (p53-GFP was a gift from Geoff Wahl (Addgene plasmid #11770)), (12091) (GFP-p53 was a gift from Tyler Jacks (Addgene plasmid #12091)) [29]. The Nedd4DN vector was a kind gift from Dr. Dale S. Haines (Temple University School of Medicine). CMV10-3xFlag Skp2 delta-F was a gift from Sung Hee Baek (Addgene plasmid # 81116) [30].

### DMBA treatments

Mice were given 1 mg of DMBA (Sigma Aldrich) in 100 μL of a sesame to corn oil mixture (4:1 ratio) via oral gavage once per week. Treatment began when mice reached 8 weeks of age and were continued for 6 consecutive weeks. Mice were monitored on a weekly basis for the presence of tumours via palpitations. Mice were humanely sacrificed when tumours were noted, and all mice were sacrificed by 8 months of age regardless of tumour formation. Tissues were collected from sacrificed mice and flash frozen for immunoblotting and qRT-PCR analysis, or fixed in formalin for immunohistochemistry. DMBA was dissolved in DMSO for all in vitro experiments and used at a final concentration of 1.5 μg/mL.

### Histology and immunostaining

Tissue was collected and fixed in 10% neutral buffered formalin. Immunohistochemistry was performed as described [31]. All primary antibodies were diluted in 3% BSA-0.1% Tween-20 in 1× PBS with the exception of mouse antibodies, which were diluted with Mouse on Mouse (MOM) blocker (Biocare Medical). Primary antibodies used were as follows: Spy1 (1:200; PA5-29417; Thermo Fisher Scientific), BrdU (1:200; 555627; BD Bioscience), γH2AX (1:200; 05-636; Millipore) Nedd4 (1:200; MBS9204431; MyBioSource), PCNA (1:500; sc-9857; Santa Cruz) and cleaved caspase-3 (1:250; 9661; Cell Signaling). Secondary antibodies were used at a concentration of 1:750 and were as follows: biotinylated anti-mouse, biotinylated anti-goat and biotinylated anti-rabbit (Vector Laboratories). Slides were imaged using the LEICA DMI6000 inverted microscope with LAS 3.6 software.

### Whole mount analysis

Briefly, the inguinal mammary gland was spread onto a positively charged slide (Fisherbrand 12-550-15) and left in Clarke’s Fluid (75% ethyl alcohol, 25% acetic acid) overnight. The following day, glands were placed in 70% ethyl alcohol for 30 min before being stained in carmine alum (0.2% carmine, 0.5% potassium aluminium sulphate) overnight. Glands were destained for 4 to 6 h with destaining solution (1% HCl, 70% ethyl alcohol) and subsequently dehydrated in ascending concentrations of alcohol (15 min each 70, 95, 100% ethyl alcohol) before being cleared in xylene overnight. Slides were mounted with Permount toluene solution (Fisher Scientific) before imaging on a Leica MZFLIII dissecting microscope (University of Windsor). Images were captured using Northern Eclipse software.

### Transfection and infection

MDA-MB-231 and MCF7 mammary cell lines were transiently transfected in serum and antibiotic-free media using 25 μg of polyethylenimine (PEI) and 12 μg of plasmid DNA, incubated at room temperature for 10 min in base media before being added to the plate. For transfection of HC11 cells, media were changed to serum and antibiotic-free media 4 h prior to transfection. After 4 h, 28 μg of PEI and 12 μg of plasmid DNA were incubated at room temperature for 10 min in base media before being added to the plate. Transfection of HEK-293 cells was performed in full growth media with 25 μg of PEI and 10 μg of plasmid DNA. For all cell lines, transfection reagent was left for 16–18 h.

Transfection of primary mouse cell lines with sip53 (Santa Cruz) and siRNA control (Santa Cruz) was performed using siRNA Transfection Reagent (Santa Cruz) as per the manufacturer’s instructions.

### UV irradiation

Media were removed from exponentially growing cells, and cells were washed once with 1× PBS and subjected to 254 nm of UV radiation using a GS Gene Linker (Bio Rad). Immediately following irradiation, fresh medium was added to the cells.

### Quantitative real-time PCR analysis

RNA was isolated using Qiagen RNeasy Plus Mini Kit as per the manufacturer’s instructions. cDNA was synthesized using Superscript II (Invitrogen) as per the manufacturer’s instructions. SYBR Green detection (Applied Biosystems) was used for real-time PCR and was performed and analysed using ViiA7 Real Time PCR System (Life Technologies) and software.

### Protein isolation and Immunoblotting

Tissue lysis buffer (50 mM Tris-HCl pH 7.5, 1% NP-40, 0.25% Na-deoxycholate, 1 mM EGTA, 0.2% SDS, 150 mM NaCl) with protease inhibitors (leupeptin 2 μg/mL, aprotinin 5 μg/mL, PMSF 100 μg/mL) was added to flash frozen tissue. Tissue and lysis buffer were homogenized on ice using a Fisher Scientific Sonic Dismembrator 50. Samples were centrifuged at 13000 rpm for 20 min at 4 °C. Supernatant was collected and centrifuged again at 13000 rpm for 20 min at 4 °C. Supernatant was collected and stored at − 20 °C until future use. Cells were lysed with TNE buffer (50 mM Tris, 150 mM NaCl, 5 mM EDTA) with protease inhibitors (leupeptin 2 μg/mL, aprotinin 5 μg/mL, PMSF 100 μg/mL). Cells were lysed for 10 min on ice and centrifuged at 4 °C at 10,000 rpm for 10 min, and supernatant was collected and stored at − 20 °C until further use.

Protein concentrations were assessed using the Bradford assay as per the manufacturer’s instructions. Equal amounts of protein were analysed and separated using SDS-PAGE and transferred to PVDF membranes. Membranes were blocked for 1 h at room temperature in 1% BSA and incubated in primary antibody overnight at 4 °C. Primary antibodies were used at a concentration of 1:1000 and were as follows: Actin (MAB1501; Millipore), p53 (ab131442; Abcam), Spy1 (ab153965; Abcam), c-Myc (C3956; Sigma Aldrich), Flag (F1804; Sigma Aldrich) and Nedd4 (MBS9204431; MyBioSource). Secondary antibody mouse or rabbit IgG (Sigma Aldrich) at a concentration of 1:10,000 was used for 1 h at room temperature. Visualization was conducted using chemiluminescent peroxidase substrate (Pierce) as per the manufacturer’s instructions. Images were captured on Alpha Innotech HD 2 using AlphaEase FC software.

### BrdU incorporation assay

Fifteen thousand cells per well were seeded in a 96-well plate. BrdU (BD Pharmingen) was added 24 h later to a final concentration of 10 μM, and cells were incubated in media containing BrdU for 24 h at 37 °C, 5% CO_2_. Media containing BrdU were removed, and cells were washed three times with 1× PBS. Cells were fixed in 4% PFA for 15 min, washed twice with 1× PBS, incubated for 20 min at 37 °C in 2 M HCl and subsequently washed once with 1× PBS. Cells were incubated for 45 min with Anti-BrdU (BD Biosciences) in 0.2% Tween in 1× PBS. Cells were washed with 1× PBS and incubated with anti-mouse IgG and Hoescht at a 1:1000 dilution in 1× PBS for 1 h at room temperature. Cells were washed one time with 1× PBS, once with distilled water, and stored at 4 °C in 50% glycerol until imaged using the LEICA DMI6000 inverted microscope.

### Flow cytometry

Mammary primary epithelial cells were isolated from inguinal glands as described [27]. Cells were stained using CD24 (APC; BD 562349) and CD45 (PeCy7; BD 552848), and FACS was performed using a BD LSR Fortessa X-20 (Becton Dickinson).

### Statistical analysis

For tumour studies, a Mann-Whitney test was performed for statistical analysis. For all other data, Student’s *T* test was performed. Unequal variance was assumed for experiments involving mouse tissue samples and primary mammary epithelial cells. Cell line data analysis assumed equal variance. All experiments, both in vitro and in vivo, included at least three biological replicates, and results are representative of at least three experimental replicates. No randomization or blinding occurred for animal studies. Significance was scored as *p < 0.05, **p < 0.01 and ***p < 0.001.

See Additional files 1, 2, 3, 4, 5 and 6 for more materials and methods.

## Results

### Generation of MMTV-Spy1 transgenic mice

**Fig. 1:**
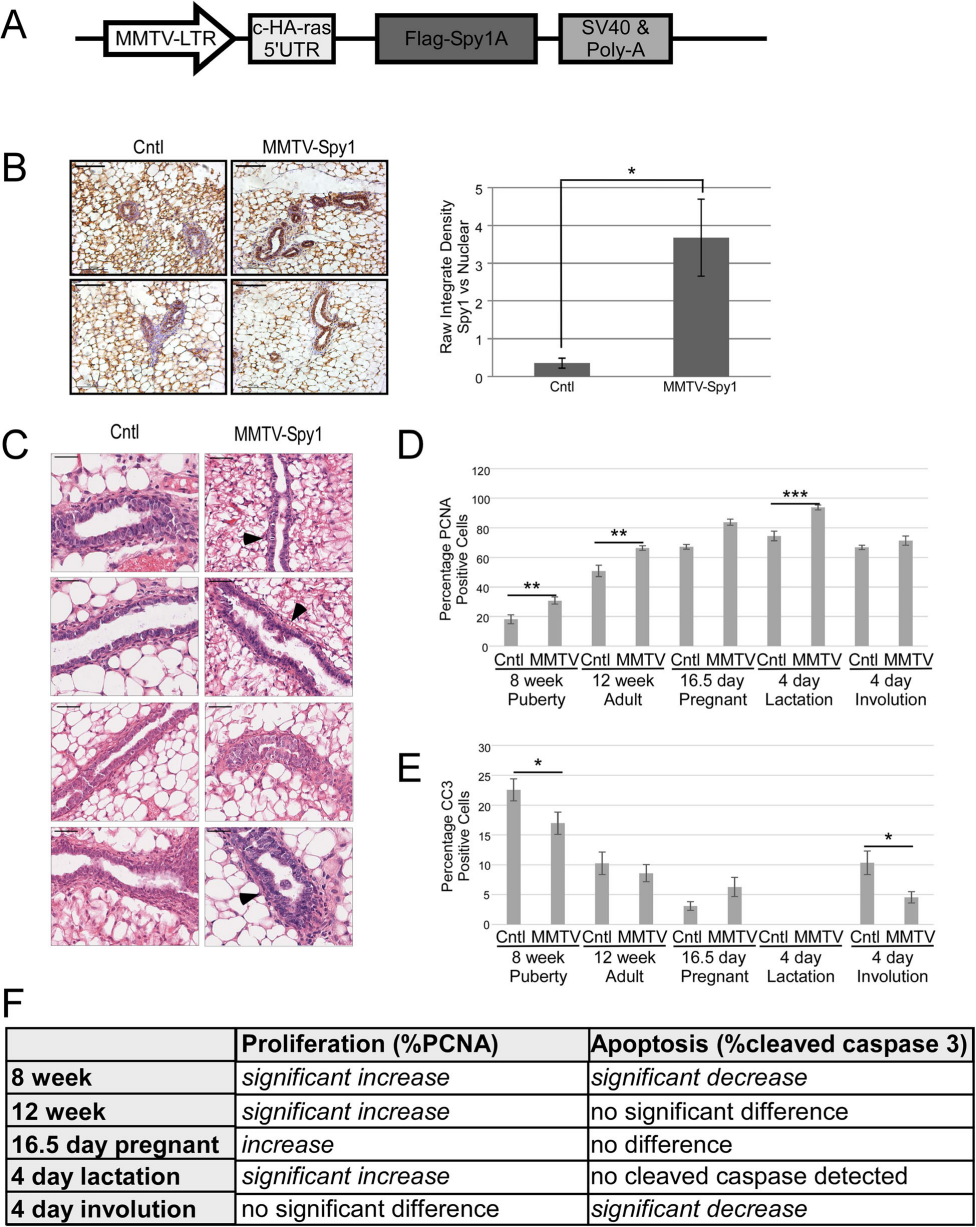
Characterization of MMTV-Spy1 mice. **a** Schematic representation of the MMTV-Spy1 transgenic vector used in pronuclear injections for the generation of the MMTV-Spy1 mouse. **b** Spy1 expression in 8-week-old MMTV-Spy1 and control littermate (Cntl) inguinal mammary glands, where blue stain is haematoxylin and brown stain represents Spy1 expression. Representative images in left panels with quantification of Spy1 levels using ImageJ software analysis shown in the right panel. Scale bar = 100 μm. **c** Representative H&E stain of inguinal mammary glands from 6-week-old MMTV-Spy1 mice and control littermates (Cntl). Arrowheads point to areas with abnormal proliferative characteristics. Scale bar = 50 μm. **d** PCNA expression in MMTV-Spy1 and littermate controls via immunohistochemical analysis. Quantification of percentage of PCNA-positive mammary epithelial cells over five fields of view per sample (8 week Cntl *n* = 3, MMTV-Spy1 *n* = 4; 12 week Cntl *n* = 3, MMT-Spy1 *n* = 3; 16.5 day pregnant Cntl *n* = 1, MMTV-Spy1 *n* = 2; 4 day lactation Cntl *n* = 3, MMTV-Spy1 *n* = 2; 4 day involution Cntl *n* = 2, MMTV-Spy1 *n* = 2). **e** Cleaved caspase-3 (CC3) expression in MMTV-Spy1 and littermate controls via immunohistochemical analysis. Quantification of percentage of CC3-positive mammary epithelial cells over five fields of view per sample (8 week Cntl *n* = 3, MMTV-Spy1 *n* = 4; 12 week Cntl *n* = 3, MMT-Spy1 *n* = 3; 16.5 day pregnant Cntl *n* = 1, MMTV-Spy1 *n* = 2; 4 day lactation Cntl *n* = 3, MMTV-Spy1 *n* = 2; 4 day involution Cntl *n* = 2, MMTV-Spy1 *n* = 2). **f** Summary of proliferation and apoptosis data for developmental time course. Error bars reflect standard error (SE), Student’s *T* test *p < 0.05, **p < 0.01, ***p < 0.001. See also Additional file 1: Figure S1 and Additional file 2: Figure S2

The flag-Spy1 coding sequence was cloned into the MMTV-SV40 plasmid (Fig. 1a) and injected into B6CBAF1/J pronuclei. PCR analysis identified three founders, with 5 to 15 copies of the transgene (data not shown), all of which successfully transmitted the transgene to their progeny (Additional file 1: Figure S1A). Analysis of both mRNA and protein levels from 6-week-old mice revealed that mammary glands from MMTV-Spy1 mice contained significantly higher levels of Spy1 as compared to control littermates (Additional file 1: Figure S1B). Western blot analysis of other tissues in the MMTV-Spy1 mice did not demonstrate significant elevation of Spy1 (Additional file 1: Figure S1C).

Previous data demonstrated that increased levels of Spy1 in immortalized ‘normal’ mouse mammary cells (HC11 cells) transplanted into cleared fat pads can disrupt morphology of the mammary gland and promote accelerated development in vivo [20]. Histopathological analysis of MMTV-Spy1 glands during puberty revealed modest phenotypic changes in the gland including a thickening of the ductal walls and some abnormal, proliferative characteristics (Fig. 1c black arrowheads). Additionally, Spy1 appeared to be expressed primarily in luminal cells and showed varying expression in myoepithelial cells (Fig. 1b, Additional file 1: Figure S1D). Flow cytometry was used to delineate between basal and luminal populations of cells as described [32] and while there does appear to be increases in epithelial content, no significant difference was observed (Additional file 1: Figure S1E). Gross morphology of the gland was not altered in whole mount analysis or histological analysis at any developmental time point analysed (Additional file 2: Figure S2A,B,C). All MMTV-Spy1 female mice successfully nursed their litters, even following multiple rounds of pregnancy, and there were no tumours noted when mice were aged for 2 years.

Spy1 increases cell proliferation in a variety of cell types when exogenously expressed [14, 22]. To determine if MMTV-Spy1 mammary glands exhibited increased rates of proliferation, immunohistochemical analysis was performed to examine the expression of PCNA throughout a developmental time course. MMTV-Spy1 mice had significantly more PCNA-positive cells than their littermate controls indicating increased proliferation at all points examined except for day 4 of involution (Fig. 1d, f, Additional file 3: Figure S3). To determine if there was a bonafide increase in proliferation with no subsequent increase in apoptosis to counterbalance enhanced proliferation, glands were analysed for expression of cleaved caspase-3. No differences in cleaved caspase-3 were detected at 12 weeks, day 16.5 pregnancy, or during lactation; however, a significant reduction in apoptosis was seen at 8 weeks and day 4 of involution (Fig. 1e, f, Additional file 3: Figure S3). This suggests that Spy1 is capable of not only enhancing proliferation but also overriding apoptosis in an in vivo setting. To further validate this finding, primary mammary epithelial cells were isolated from the inguinal mammary glands of control and MMTV-Spy1 mice and treated with BrdU. Cells from MMTV-Spy1 inguinal mammary glands were found to have a significantly higher percentage of BrdU-positive cells (Additional file 2: Figure S2D). Hence, MMTV-Spy1 mice display modest phenotypic and no gross morphological changes in the mammary gland despite having enhanced proliferation and decreased apoptosis.

### Spy1 increases mammary tumour susceptibility

**Fig. 2:**
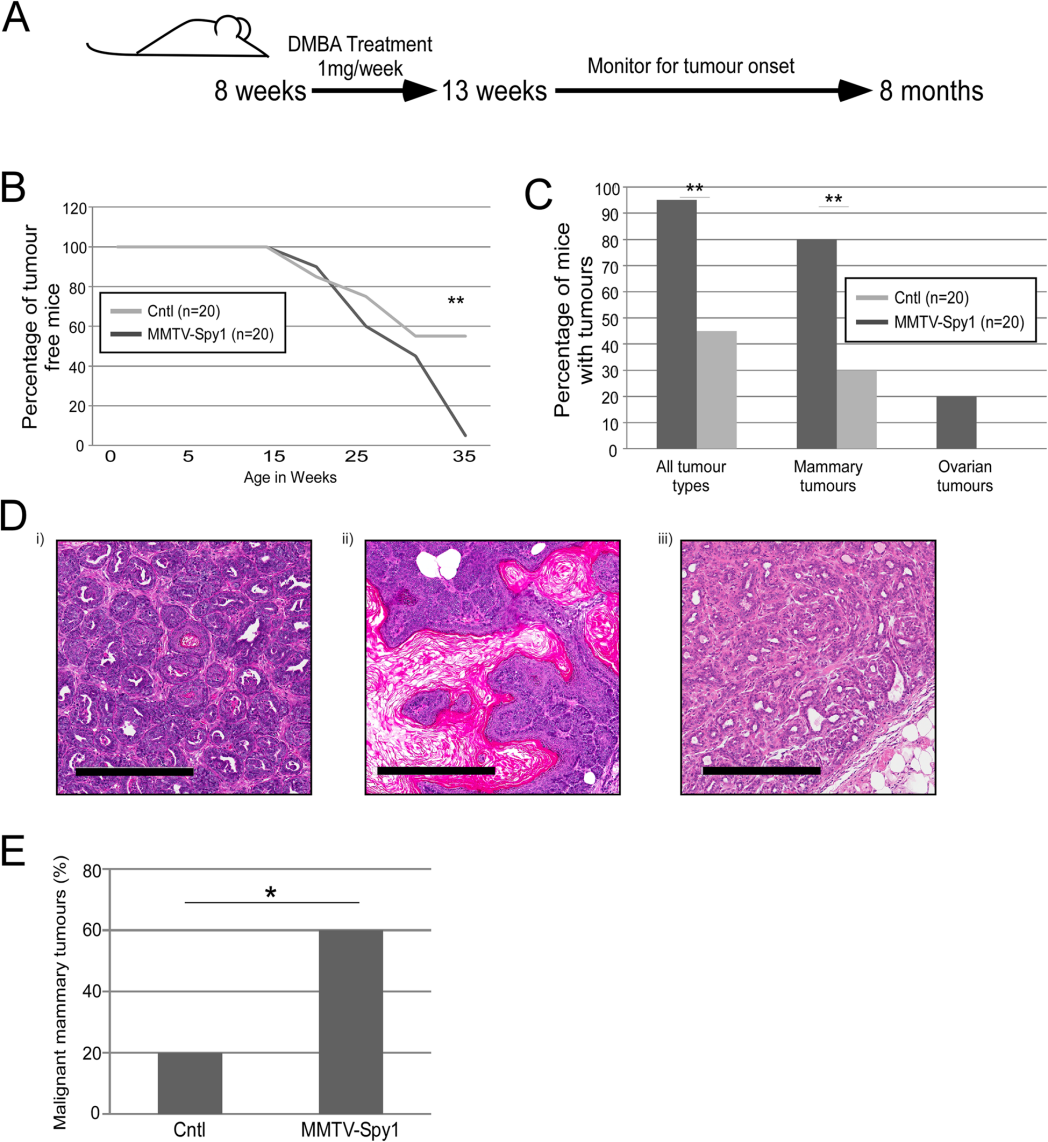
Spy1 overexpression leads to increased mammary tumour susceptibility. **a** Schematic of DMBA treatment. **b** Graphical representation of timing of tumour onset (*n* = 20). **c** Graphical representation of percentage of mice with tumours (*n* = 20). **d** Representative images of tumour pathology from DMBA-induced mammary tumours (i), adenosquamous carcinoma, (ii) and adenomyoepithelioma (iii). Scale bar = 300 μm. **e** Graphical representation of the number of mice with malignant mammary tumours (*n* = 20). Mann-Whitney *p < 0.05, **p < 0.001

Although MMTV-Spy1 mammary glands exhibit significant changes in proliferative capacity, they develop normally and do not present with spontaneous tumours. Increased protein levels of Spy1 have been implicated in several human cancers including that of the breast, ovary, liver and brain [20, 22–24]. To assess whether or not elevated levels of Spy1 may affect tumour susceptibility, MMTV-Spy1 mice and control littermates were treated with the mammary carcinogen 7,12-dimethlybenz(a) anthracene (DMBA) once per week for 6 consecutive weeks during puberty (Fig. 2a). DMBA induces DNA damage through the formation of DNA adducts and is commonly used in rodent models to study the onset and timing of mammary tumour formation [33–35]. Mice were monitored on a weekly basis for tumour formation. The timing of tumour initiation was not altered (Fig. 2b); however, 95% of MMTV-Spy1 mice developed tumours as compared to only 45% of control mice (Fig. 2c). Of the tumours developed, 80% of MMTV-Spy1 mice presented with mammary tumours both benign and malignant, as compared to only 30% of littermate controls. Interestingly, ovarian tumours occurred in MMTV-Spy1 mice, but there was no incidence of ovarian tumours in littermate controls. Tumour tissue was sent for pathological analysis, and MMTV-Spy1 mice had significantly more malignant mammary tumours over littermate controls (Fig. 2d, e).

### p53 can regulate protein levels of Spy1

**Fig. 3:**
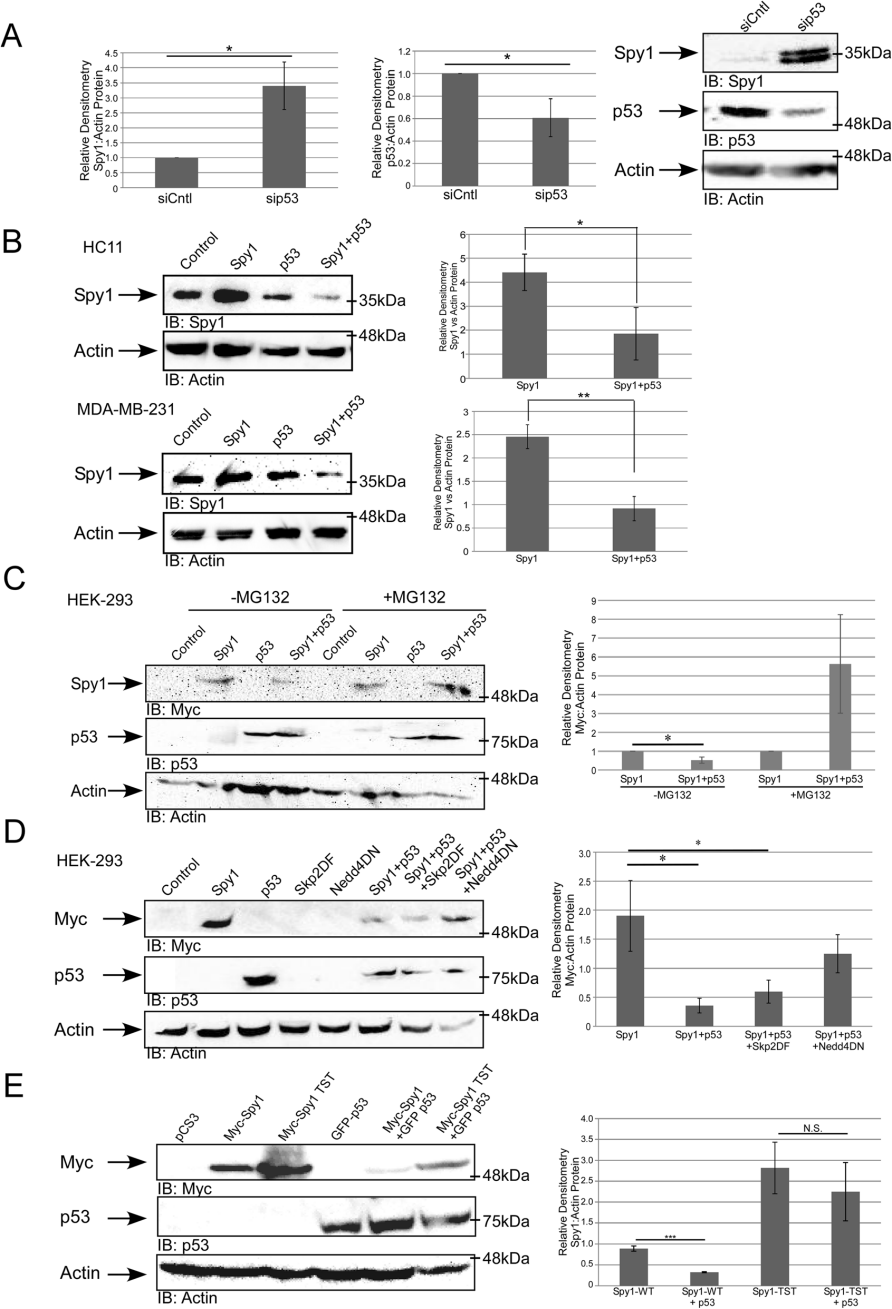
p53 regulates Spy1 protein levels through the ubiquitin ligase Nedd4. **a** Western blot analysis of Spy1 (left panel) and p53 (middle panel) protein levels in MMTV-Spy1 primary mammary epithelial cells corrected for Actin. Data is represented as a fold change as compared to control siRNA (siCntl). Representative blot is shown in the right panel. **b** Levels of Spy1 protein were assessed via Western blot analysis 24 h after transfection in HC11 (*n* = 6) and MDA-MB 231 (*n* = 5) cells transfected with pEIZ, pEIZ-Spy1, p53 or both pEIZ-Spy1 and p53. Left panels depict representative blots and right panels depict densitometry analysis of Spy1 levels corrected for Actin. **c** Levels of Spy1 protein were assessed via Western blot analysis in the presence and absence of MG132. The left panel depicts representative blot, and the right panel depicts densitometry analysis of Spy1 protein levels corrected for Actin. Data is shown as fold change to cells transfected only with the Spy1 vector (*n* = 3). **d** Levels of Spy1 protein were assessed in HEK-293 cells after transfections with control vector pCS3 and Myc-Spy1-pCS3, p53, Skp2ΔF and Nedd4DN in various combinations. Cells were collected 24 h after transfection and subjected to Western blot analysis. Densitometry analysis was performed for total Spy1 protein levels and corrected for total Actin levels (*n* = 3). **e** Levels of Spy1 and Spy1-TST protein were assessed in HEK-293 cells after transfection with control vector pCS3, myc-Spy1-pCS3, myc-Spy1-TST-pCS3 and p53. Cells were collected 24 h after transfection and subjected to Western blot analysis. Densitometry analysis was performed for total Spy1 protein levels and corrected for total Actin levels (*n* = 3). Error bars represent SE; Student’s *T* test. *p < 0.05, **p < 0.01, ***p < 0.001, not significant (N.S.). See also Additional file 4: Figure S4

Previous mammary fat pad transplantation of Spy1 overexpressing HC11 cells leads to increased tumour formation in vivo [20]. HC11 is an immortalized cell line with mutated p53 that renders p53 non-functional [36–38]. Spy1 is capable of preventing checkpoint activation [15], and since p53 plays a critical role in mediating proper checkpoint activation, it is plausible then that the lack of spontaneous tumours in the MMTV-Spy1 mice may be attributed to the presence of wild-type p53. To test this theory, primary mammary epithelial cells were extracted from an MMTV-Spy1 mouse and p53 was knocked down using siRNA (Fig. 3a). Interestingly, with only a modest decrease in p53 protein levels (Fig. 3a; middle panel), there was a very significant increase in Spy1 protein levels (Fig. 3a; left panel). Given that tumour formation was seen in a cell line with non-functional p53, and Spy1 can prevent checkpoint activation [13, 15, 16, 20], it is plausible then that wild-type p53 may work to downregulate Spy1 to allow for p53-mediated cell cycle arrest, and elevated Spy1 with loss of p53 function would allow for enhanced genomic instability. To test the ability of wild-type p53 to regulate levels of Spy1 protein, mammary cells with mutated p53 (HC11 and MDA-MB-231 cells) were transfected with pEIZ, pEIZ-Spy1, p53 or pEIZ-Spy1 and p53 and lysates collected at 24 h for Western blot analysis. Levels of Spy1 protein were significantly decreased in the presence of wild-type p53 (Fig. 3b). This result was also seen in an additional two cell lines (data not shown). To determine if p53 also affected Spy1 mRNA, MDA-MB-231 cells were transfected with pEIZ, pEIZ-Spy1, p53 or pEIZ-Spy1 and p53 and levels of mRNA were assessed via qRT-PCR. There was no effect on levels of Spy1 mRNA in the presence of elevated p53 indicating that p53 likely regulates Spy1 expression at the level of protein expression (Additional file 4: Figure S4A). Previous data has demonstrated that Spy1 is targeted for proteasome-dependent degradation in different phases of the cell cycle, with a dependency on the E3 ubiquitin ligase Nedd4 in G2 [28] and on the Skp2 ubiquitin ligase in G1 [39]. To first determine if the downregulation of Spy1 by p53 is proteasome dependent, Spy1 and p53 were expressed in the presence of the proteasome inhibitor MG132. Inhibition of the proteasome in the presence of p53 abrogated the downregulation of Spy1 protein, supporting that p53 regulates protein levels of Spy1 via a proteasome-dependent mechanism (Fig. 3c). To further determine whether this effect is dependent on classically defined E3 ligases targeting Spy1, Spy1 and p53 were overexpressed along with dominant-negative forms of both Nedd4 and Skp2. Levels of Spy1 were significantly decreased in the presence of p53 and the dominant-negative Skp2; however, loss of Nedd4 activity significantly reduced the ability of p53 to decrease levels of Spy1 (Fig. 3d). To determine if p53 is capable of mediating levels of Nedd4, p53 was overexpressed and protein and RNA levels of Nedd4 were examined. No significant differences were seen at either the levels of protein or RNA (Additional file 4: Figure S4B,C). Previous data has also demonstrated that post-translational modification of Spy1 at residues Thr15, Ser22 and Thr33 targets Spy1 for degradation by Nedd4 [28]. Wild-type Spy1 and a mutant non-degradable by Nedd4 (Spy1-TST) were both overexpressed in the presence of p53. Levels of wild-type Spy1 are significantly decreased in the presence of p53; however, p53 is unable to downregulate Spy1-TST indicating that post-translational modifications of Spy1 play an important role in p53-mediated degradation of Spy1 (Fig. 3e). Spy1-TST was also not degraded following exposure to UV in opposition to wild-type Spy1, which is significantly decreased following UV damage (Additional file 4: Figure S4D). This data supports that Spy1 levels are tightly controlled by p53 and this response is dependent on classically defined ubiquitin-mediated mechanisms for Spy1.

### Spy1 downregulation is a necessary component of the DDR

**Fig. 4:**
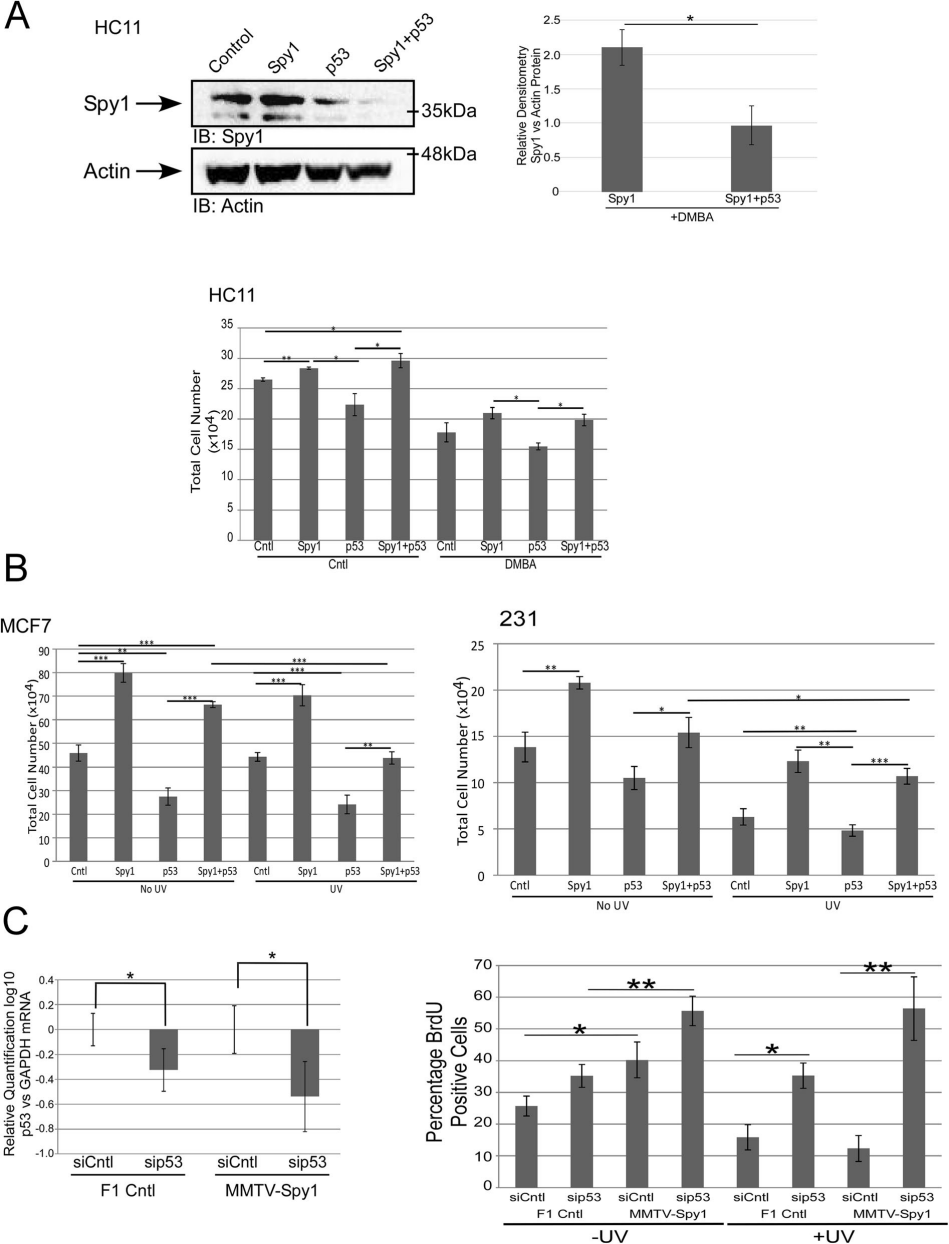
Spy1 can enhance proliferation in the presence of p53. **a** HC11 cells were transfected with vector control, pEIZ-Spy1, p53 or pEIZ-Spy1 and p53 in the presence or absence of 1.5 μg/mL of DMBA. Levels of Spy1 are depicted (upper panels). Growth of cells following transfection was assessed via trypan blue analysis (lower panels) (*n* = 3). **b** MCF7 (left panel) and MDA-MB 231 (right panel) were transfected with vector control, pEIZ-Spy1, p53 or pEIZ-Spy1 and p53 in the presence or absence of 50 J/m^2^ UV damage. Growth of cells following transfection was assessed via trypan blue analysis (*n* = 3). **c** qRT-PCR analysis of p53 levels in littermate control (F1 Cntl) and MMTV-Spy1 primary mammary epithelial cells corrected for total GAPDH (left panel). Quantification of BrdU-positive cells with and without UV irradiation with (siCntl) and without p53 (sip53) (right panel). F1 Cntl *n* = 5, MMTV-Spy1 *n* = 5. Error bars represent SE; Student’s *T* test. *p < 0.05, **p < 0.01, ***p < 0.001

Spy1 can override the function of downstream effectors of p53 [13, 15]; hence, we hypothesize that negative regulation of Spy1 by wild-type p53 may be essential to ensure a healthy DDR response. To test this, cell proliferation was measured in HC11, MCF7 and MDA-MB-231 cells following Spy1, p53 or Spy1 and p53 overexpression in the presence or absence of DNA damage stimuli (Fig. 4a, b). Spy1 was capable of overriding the effects of constitutive expression of p53 both in the presence and absence of damage in both DMBA (Fig. 4a) and UV damage (Fig. 4b). It is notable that this effect was independent of endogenous p53 status. To further examine the functional relationship between Spy1 and p53 in primary mammary epithelial cells, p53 levels were manipulated with siRNA in cells extracted from the MMTV-Spy1 mice or littermate controls (Fig. 4c; left panel). Cell proliferation was measured in the presence and absence of UV damage (Fig. 4c; right panel). These data demonstrate that endogenous levels of wild-type p53 keep a check on primary mammary populations in both the presence and absence of damage and that loss of p53 resulted in a robust increase in Spy1-mediated effects on proliferation.

### Spy1 expression disrupts the DDR in the presence of DMBA

**Fig. 5:**
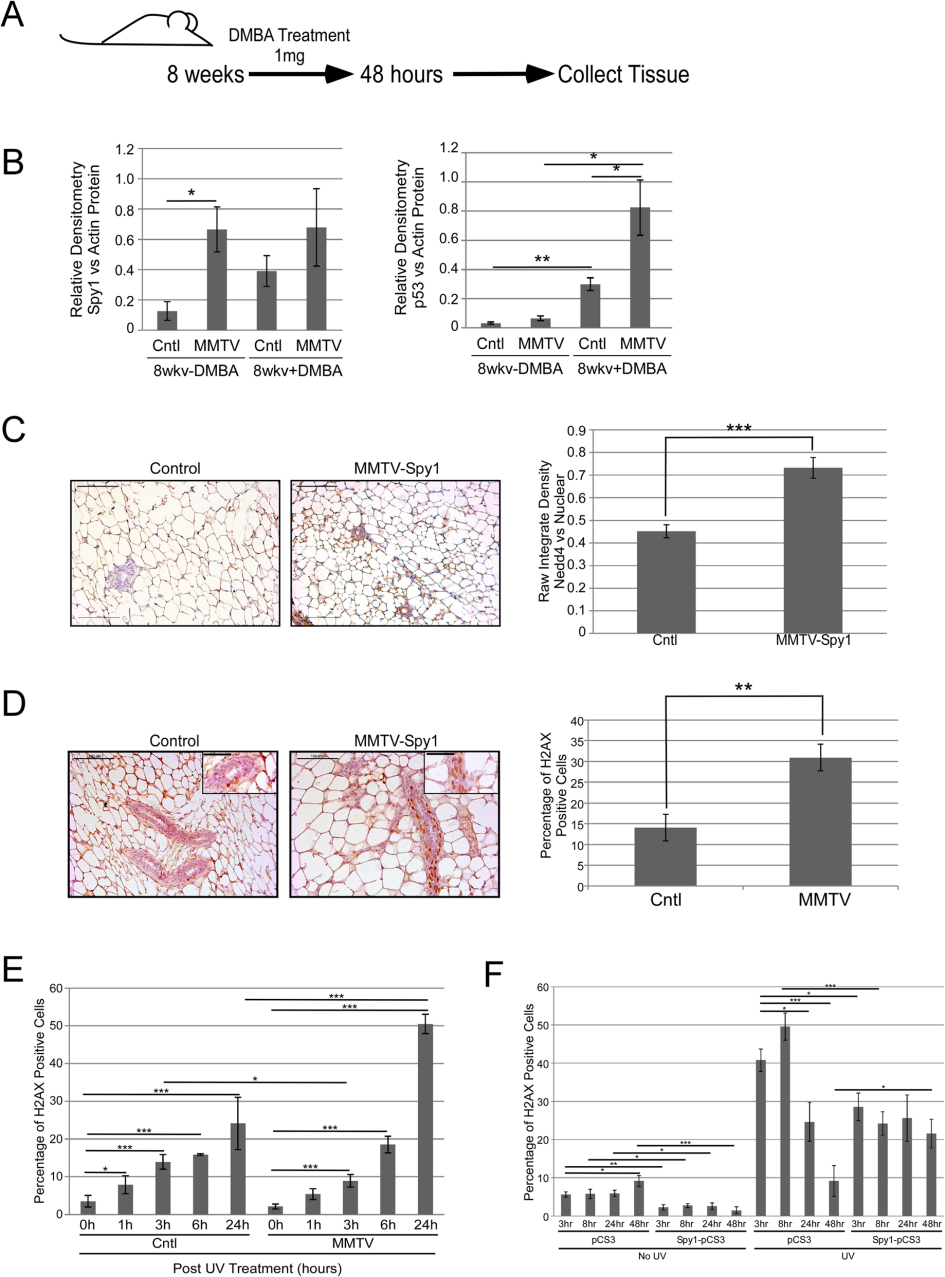
MMTV-Spy1 mice show alterations in DDR pathway when exposed to DMBA. **a** Schematic of short-term DMBA treatment and collection of samples. **b** Western blot for Spy1 (left panel) and p53 (right panel) levels in 8-week-old control mice and DMBA-treated mice 48 h following DMBA exposure. Densitometry analysis is depicted with total Spy1 and p53 levels corrected for total levels of Actin. **c** Immunohistochemical analysis for Nedd4 expression in inguinal mammary glands of 8-week-old MMTV-Spy1 mice and littermate controls was performed after exposure to DMBA. Representative images are shown in the left panel. Levels of Nedd4 were quantified using ImageJ analysis (right panel). Scale bar = 100 μm. **d** Representative images of immunohistochemical analysis of γH2AX in inguinal mammary glands of 8-week-old MMTV-Spy1 and littermate control (Cntl) mice after exposure to DMBA (left panel), where brown stain is γH2AX and blue stain is haematoxylin. Number of γH2AX-positive cells were counted and quantified as percentage of γH2AX cells (right panel). Scale bars = 100 μm and 50 μm (inset image). **e** Primary mammary epithelial cells from MMTV-Spy1 mice and control littermates were isolated and UV irradiated with 50 J/m^2^. Cells were collected 0, 1, 3, 6 and 24 h post UV, and immunofluorescence was performed to assess formation of γH2AX foci following damage (*n* = 3). **f** HC11 cells were transfected with pCS3 and Myc-Spy1-pCS3, and UV irradiated with 50 J/m^2^. Cells were analysed at various times following UV irradiation for the number of γH2AX-positive cells via immunofluorescence. Error bars represent SE; Student’s *T* test. *p < 0.05, **p < 0.01, ***p < 0.001

To validate the in vitro findings that Spy1 elevation can alter proper checkpoint activation, MMTV-Spy1 mice were treated with 1 mg DMBA, and inguinal mammary gland tissues were collected after 48 h and analysed for alterations in known DDR proteins (Fig. 5a). Spy1 was significantly overexpressed at the mRNA level in 8-week-old MMTV-Spy1 mice with and without DMBA (Additional file 5: Figure S5A). Spy1 protein levels were also elevated in the MMTV-Spy1 mice over littermate controls both in the presence and absence of DMBA (Fig. 5b; left panel). Importantly, Spy1 protein levels increased in control mice following treatment with DMBA in accordance with previous data demonstrating Spy1 is upregulated in response to damage [15]. Interestingly, p53 levels were significantly higher in the MMTV-Spy1 mice over littermate controls after DMBA treatment (Fig. 5b compare left to right panels, Additional file 5: Figure S5B). MMTV-Spy1 mice treated with DMBA were also found to have a significant increase in Nedd4 expression at the same time as p53 suggesting an upregulation in pathways responsible for Spy1-mediated degradation (Fig. 5c).

### Elevated levels of Spy1 lead to accumulated DNA damage

The effects of Spy1 on the level of DNA damage following exposure to DMBA was investigated in vivo. MMTV-Spy1 mice at 8 weeks of age were again treated once with DMBA, and samples were collected and analysed 48 h post treatment. MMTV-Spy1 mice had significantly more γH2AX-positive cells as compared to littermate controls, indicating a lack of repair in response to DMBA (Fig. 5d). To determine if this is ubiquitous for different forms of DNA damage, primary inguinal mammary gland cells from MMTV-Spy1 mice and control littermates were isolated and UV irradiated with 50 J/m^2^. Expression of γH2AX was monitored at a time course following damage. Cells from MMTV-Spy1 mice had significantly more γH2AX-positive cells at 24 h post UV as compared to control littermate cells (Fig. 5e). Data from the MMTV-Spy1 mouse both in vivo and in vitro shows a significant increase in γH2AX following DNA damage, which is in opposition to previously published data, which shows a significant decrease in γH2AX with Spy1 overexpression [13, 16]. To determine if this is due to a difference in the time points studied, HC11 cells were transfected with pCS3 or Myc-Spy1-pCS3, UV irradiated and studied at a wide time course. At all times collected in non-irradiated cells, Spy1 overexpression led to a significant decrease in γH2AX as compared to control (Fig. 5f). Following UV, however, γH2AX was significantly lower in Spy1 cells at early time points and then significantly higher at 48 h post UV. Previous work has examined the role of Spy1 in checkpoint activation following damage [13, 16]. Spy1 overexpression leads to decreased activation of both S phase and G2M checkpoints, as well as decreased activation of DDR signalling as assessed through Chk1 phosphorylation status [13, 16]. Spy1 also decreased rates of removal of damage following UV, indicating that elevated levels of Spy1 prevent cellular checkpoint activation and impair removal of damage [13]. This data supports that elevated levels of Spy1 may promote proliferation and a delayed or impaired recognition of DNA damage at early time points; however, overriding checkpoints over time leads to an accumulation of DNA damage.

### In the absence of p53, Spy1 drives hyperplasia

**Fig. 6:**
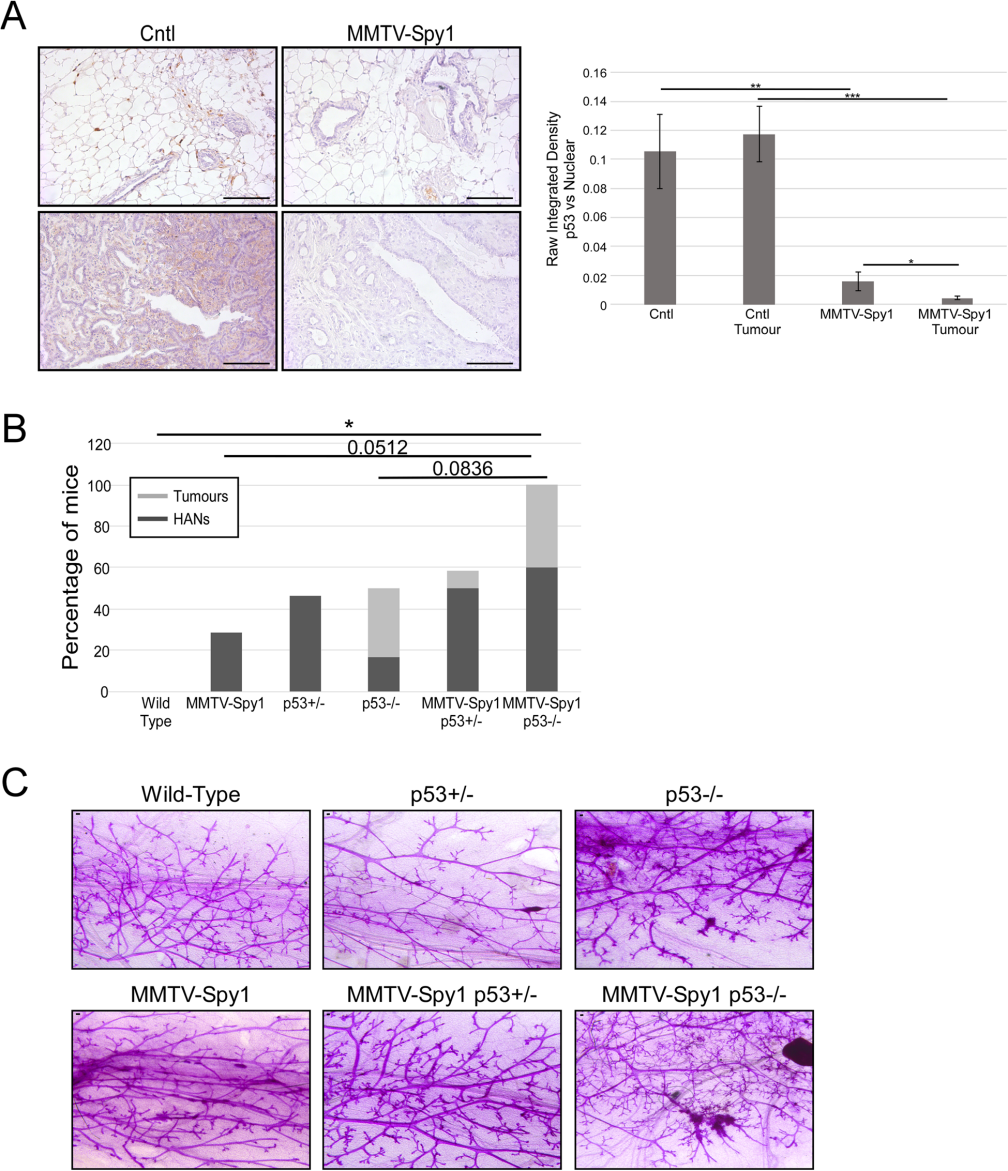
Loss of p53 enhances hyperplasia in MMTV-Spy1 mice. **a** Immunohistochemical analysis for p53 expression in inguinal mammary glands and tumours of DMBA-treated MMTV-Spy1 mice and littermate controls. Representative images are shown in the left panel. Levels of p53 were quantified using ImageJ analysis (right panel). Scale bar = 100 μm. **b** Fat pads of wild-type mice were reconstituted with mammary epithelial cells from MMTV-Spy1 mice crossed with p53 null mice and were monitored for HANs and formation of tumours. Only tumour-negative mice were screened for the formation of HANs (wild-type *n* = 5; MMTV-Spy1 *n* = 7, p53+/− *n* = 13; p53−/− *n* = 6; MMTV-Spy1 p53+/− *n* = 12; MMTV-Spy1 p53−/− *n* = 5). **c** Representative images of whole mounts. Scale bar = 0.1 mm. Error bars represent SE; Student’s *T* test (**a**) and Mann-Whitney (**b**). *p < 0.05, **p < 0.01, ***p < 0.001

To determine if loss of p53 cooperates with Spy1 to promote tumourigenesis, levels of p53 were assessed in DMBA-treated MMTV-Spy1 mice and their control littermates at end point (Fig. 2a). Levels of p53 were significantly lower in both MMTV-Spy1 DMBA-induced mammary tumours as well as surrounding normal mammary tissue as compared to control (Fig. 6a). Interestingly, there was no difference in p53 expression in control surrounding normal mammary tissue as compared to control DMBA mammary tumours, while MMTV-Spy1 DMBA mammary tumours had significantly lower p53 as compared to MMTV-Spy1 normal mammary tissue (Fig. 6a). Next, MMTV-Spy1 mice were intercrossed with p53 null mice. First, inguinal mammary glands were collected from 8-week-old mice from the resulting crosses to validate earlier findings that loss of p53 leads to increased Spy1 expression (Fig. 3a). Immunohistochemical analysis shows an approximate 3.7-fold increase in Spy1 protein levels in p53 heterozygote mice as compared to wild-type mice (Additional file 6: Figure S6A), which validates the findings in Fig. 3a. To determine if Spy1 cooperates with loss of p53 to enhance proliferation, PCNA staining was performed on 8-week-old inguinal mammary glands from intercrossed MMTV-Spy1 and p53 null mice. MMTV-Spy1 alone showed a significant increase in PCNA-positive cells, and loss of one allele of p53 was sufficient to significantly increase the percentage of PCNA-positive cells over that of control mice (Additional file 6: Figure S6B). Importantly, the addition of Spy1 elevation with loss of one allele of p53 significantly enhanced proliferation over p53 heterozygotes alone (Additional file 6: Figure S6B). Mammary fat pad transplantation was performed when mice were 8 weeks of age to transplant extracted primary mammary epithelial cells from the resulting crosses into the cleared fat pad of 3-week-old wild-type mice to eliminate the possibility of other tumours forming prior to the onset of mammary tumours. Mice were left to age for up to 2 years and monitored for formation of spontaneous mammary tumours. Whole mount analysis was performed on glands that did not develop tumours to assess for the formation of hyperplastic alveolar nodules (HANs) (Fig. 6b, c). There was a significant increase in formation of HANs and tumours in fat pads of wild-type mice reconstituted with mammary epithelial cells from intercrossed MMTV-Spy1 p53−/− mice as compared to mice reconstituted with wild-type mammary epithelial cells. One MMTV-Spy1 p53+/− mouse developed a mammary tumour at 25 weeks post-transplant, while no p53+/− mice developed tumours even when left to 2 years of age. Two p53−/− and two MMTV-Spy1 p53−/− mice developed tumours, and there was no difference in the number of glands with HANs or tumours when comparing p53+/− to MMTV-Spy1 p53+/−. Complete loss of p53 with elevated levels of Spy1 leads to increased formation of HANs when comparing p53 loss alone with p53 loss combined with elevated Spy1 (Fig. 6b). Numbers of both p53 −/− and MTMV-Spy1 p53−/− were lower than expected Mendelian ratios likely due to embryonic lethality. Elevated levels of Spy1 appear to enhance hyperplastic growth of mammary gland tissue when combined with loss of p53. This data supports the conclusion that wild-type p53 holds Spy1 levels in check to permit successful checkpoint regulation and preserve genomic integrity of the gland.

## Discussion

Development of the transgenic MMTV-Spy1 mouse has yielded new insight into the molecular regulation of the breast during development, revealing how misregulation of cell cycle checkpoints can impact susceptibility to tumourigenesis. On the tumour-resistant B6CBAF1/J background, the MMTV-Spy1 mice develop normally, showing no overt phenotypic differences and no spontaneous tumourigenesis, despite a significant increase in proliferative potential of mammary epithelial cells [40]. Primary mammary epithelial cells also demonstrate increased proliferative potential. Previous data demonstrated that overexpression of Spy1 in the murine HC11 cell line shows disrupted two-dimensional acinar development in vitro, accelerated ductal development in vivo and increased tumourigenesis when transplanted into cleared mammary fat pads [20]. One difference between these systems is the HC11 cell line contains a mutated p53 which renders p53 non-functional [36–38]. Investigating this hypothesis, we found that the knockdown of p53 in MMTV-Spy1 primary mammary epithelial cells increases Spy1 protein levels significantly. To examine the relationship between Spy1 and p53, we turned our attention to in vitro cell systems, using a variety of cell lines differing in the status of p53 and DNA repair pathways. We found an inverse relationship between Spy1 and p53 protein levels in every cell system studied, and constitutive induction of Spy1 was capable of abrogating p53-mediated effects on proliferation in all scenarios. This supports previous functional data demonstrating that Spy1 can override the DDR and bypass checkpoint responses [12, 13, 15, 16]. Importantly, previous work has shown that a decrease in Spy1 leads to decreased rates of proliferation and increased apoptosis and triggers an intrinsic DDR [13, 14, 24, 41]. This demonstrates that the loss of Spy1 may actually sensitize cells to a growth arrest and the DDR. We also demonstrated that p53-mediated degradation of Spy1 is proteasome dependent and under these treatment conditions requires the E3 ligase Nedd4. p53 was unable to degrade a non-degradable mutant of Spy1, Spy1-TST, demonstrating the importance of post-translational modifications in this process. Nedd4 is unable to degrade Spy1-TST during G2/M phase of the cell cycle, and Spy1-TST is able to enhance foci formation and mammary tumourigenesis [28]. We cannot rule out that cell cycle dynamics under these treatment conditions depend more on the G2-mediated mechanism of degradation. This work does however demonstrate the importance of classically defined pathways of Spy1 degradation in maintaining the integrity of cellular checkpoints to prevent the onset of tumourigenesis. Collectively, these data support that p53 targets Spy1 protein levels to ensure the normal functioning of the DDR.

Mice treated with DMBA had elevated p53 levels, along with a significant increase in the number of γH2AX cells. The elevated p53 seen in the MMTV-Spy1 mice upon exposure to DMBA without the subsequent decrease in Spy1 levels shown in cell systems may be due to the strong viral promoter in the transgene which would allow for consistent elevation of Spy1 despite the mounting p53 response to try and decrease levels. Increased levels of γH2AX can signify latent unrepaired damage, or perhaps a delay in the repair response to DNA damage. Increased expression of γH2AX is indicative of increased levels of DNA damage, which in turn can lead to accumulation of deleterious mutations and onset of tumourigenesis. Alterations in the accumulation and subsequent decrease in γH2AX are also shown in vitro indicating alterations to the DNA damage response. We demonstrate that indeed the MMTV-Spy1 mice present with a significant increase in tumour formation. While there were some interesting findings with the histology of DMBA-induced tumours, no significant differences were found between DMBA-induced tumours in control versus MMTV-Spy1 mice. Many of the histologies noted are commonly found in DMBA-induced tumours; however, further investigation is warranted to determine if Spy1 is capable of driving different subtypes or histologies of breast cancer [42, 43].

When crossed with p53 null mice, fat pads of wild-type mice reconstituted with mammary epithelial cells from intercrossed MMTV-Spy1 mice with loss of p53 had more hyperplasia and tumours over wild-type mice reconstituted with wild-type mammary epithelial cells. The data suggests that complete loss of p53 may enhance the ability of Spy1 to drive tumourigenesis. To test this, MMTV-Spy1 primary mammary epithelial cells were manipulated for p53 levels and data supports this hypothesis; there is a significant increase in proliferation in the absence of p53. Future work to combine this with known oncogenic drivers is an important next step. Reports in the literature show the loss of p53 alone on a susceptible strain of mouse leads to formation of mammary tumours in 75% and 55% of p53 null and heterozygous mice respectively [44]. It is important to note the differences in strain between the reported literature and the MMTV-Spy1 and p53 intercross described in this study. While BALB/c mice are known to be more susceptible to mammary tumour formation, C57BL/6 mice are known to be more resistant, which may also account for lower rates of tumour onset seen with the MMTV-Spy1 and p53 null intercross [40, 45]. Given that the MMTV promoter has mosaic expression, a whole body p53 knockout was used to increase targeting in the same cells. Our data supports that elevated protein levels of Spy1 cooperate with these events.

Increased susceptibility to breast cancer, such as familial cases of breast cancer, is often caused by inherited mutations in genes that regulate the DDR, such as BRCA and p53 [5, 11, 46, 47]. It is likely that other genes which mediate cell cycle progression and alter the DDR may also be involved in enhanced susceptibility. Interestingly, studies investigating genes involved in breast cancer susceptibility have identified chromosome 2p, and specifically 2p23.2, as a site which may have genes that contribute to increased breast cancer risk [48–50]. This identified location maps directly to the chromosomal location of the Spy1 gene (*SPDYA*). While further work is needed to definitively identify Spy1 as a breast cancer susceptibility gene, the current data provides support for Spy1 in enhancing susceptibility.

## Conclusions

**Fig. 7:**
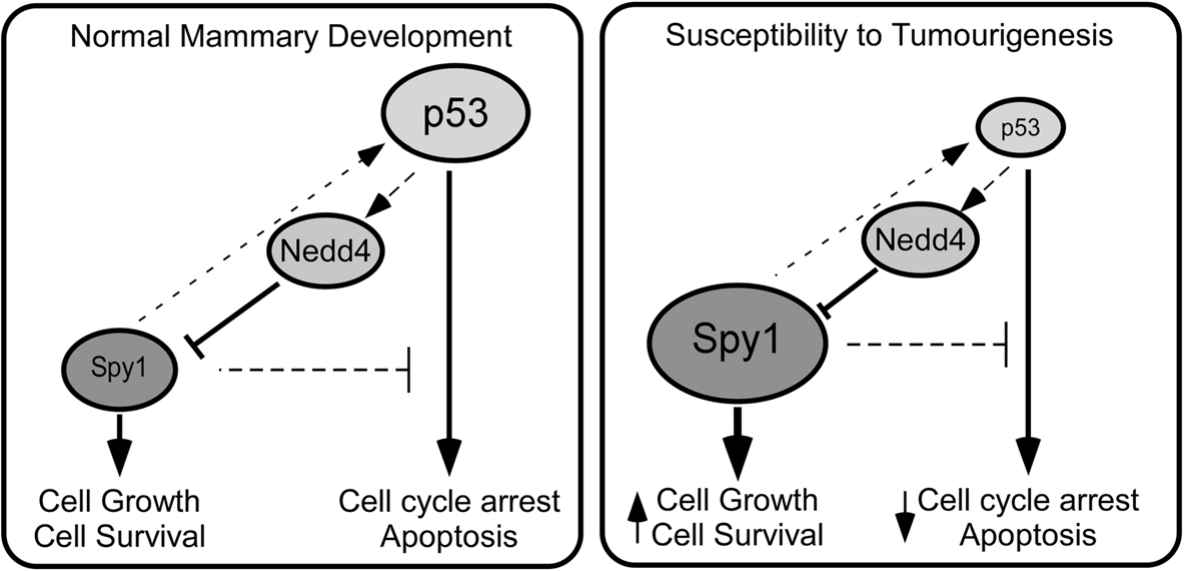
Mechanism for increased susceptibility by elevation of Spy1. The left panel reflects that Spy1 protein levels are held in check by wild-type p53 to allow tightly regulated bursts of needed mammary proliferation during development. The panel to the right reflects the situation when either p53 is mutated/deleted or Spy1 protein levels are elevated, supporting susceptibility to tumourigenesis

Collectively, this work presents a novel feedback loop between the atypical cell cycle regulator Spy1 and the tumour suppressor protein p53, where tight control over Spy1 protein levels is required to maintain normal expansion of the developing mammary epithelium. When p53 is mutated, or Spy1 is expressed at elevated levels, this will allow for deleterious mutations to accumulate, increasing susceptibility to tumourigenesis (Fig. 7). Restoring p53 function has been an elusive target in the clinic. Spy1-Cdk regulation is a unique and potentially potent mechanism for drug design, which may represent a novel therapeutic approach for select forms of breast cancer.

## Supplementary information

**Additional file 1: Figure S1.** Related to Figure 1: Characterization of MMTV-Spy1 mouse model system. A) Blots showing PCR analysis to confirm presence of transgene in founder mice (upper blot) and in offspring from founders (lower blot) where V represents vector control, V10 represents 10 copy vector control and V1 represents 1 copy vector control. B) qRT-PCR analysis of MMTV-Spy1 and littermate control (cntl) inguinal gland samples for Spy1 levels corrected for total levels of GAPDH. (n=8; left panel). Levels of Spy1 protein in 6-week-old MMTV-Spy1 inguinal glands were quantified and corrected for total actin protein levels (right panel) (n=8). C) Quantification of densitometry analysis of Spy1 protein levels corrected for total actin levels (right panel). (Salivary gland n=3; Spleen n=6). D) Representative images of Spy1 immunohistochemical analysis in MMTV-Spy1 and littermate control 8-week-old mice showing Spy1 localization within the mammary gland. Scale bar=50 μm. E) Flow cytometry of primary cells extracted from MMTV-Spy1 and littermate controls and stained for CD24 and CD45. Fold change in myoepithelial (CD24loCD45-) and luminal (CD24hiCD45-) cell population is depicted (n=3). Error bars reflect SE. Student’s T-test *p<0.05, **p<0.01.

**Additional file 2: Figure S2.** Related to Figure 1: Analysis of MMTV-Spy1 early development. A) Representative images of whole mount analysis from 6-week-old MMTV-Spy1 and littermate control (cntl) B6CBAF1/J mice (Cntl n=4, MMTV-Spy1 n=4). B) Graphical representation of analysis of whole mount images from B6CBAF1/J inguinal glands from MMTV-Spy1 and littermate controls. Number of side branches per gland was quantified and the average number of side branches per gland was calculated (left panel). Ratio of ductal progression of ductal network past the lymph node was measured for each gland, and the average rate of ductal progression is shown in the right panel. C) Representative hematoxylin and eosin images from i) 16.5 day pregnancy, ii) 4 day lactation and iii) 4 day involution. Scale bar= 100 μm. D) Representative images (right panel) and quantification of BrdU incorporation (left panel) of primary mammary epithelial cells isolated from MMTV-Spy1 mice and their control littermates (cntl) (n=4 separate isolations). Scale bar= 50 μm. Error bars represent SE. *p<0.05.

**Additional file 3: Figure S3.** Related to Figure 1: Spy1 increases proliferation during development in mammary epithelial cells. Representative images are shown of A) PCNA and B) cleaved caspase 3 at i) 8 week puberty, ii) 12 week adult, iii) 16.5 day pregnancy, iv) 4 day lactation and v) 4 day involution. Arrowheads point to A) PCNA and B) cleaved caspase 3 positive cells. Scale bar= 100 μm.

**Additional file 4: Figure S4.** A) Spy1 and p53 were overexpressed in MDA-MB-231 cells to determine if p53 can alter Spy1 mRNA levels (*n* = 3). B & C) p53 or control vector was overexpressed in HEK-293 cells to assess protein and RNA levels of Nedd4. B) Western blot analysis of Nedd4 protein levels corrected for total Actin protein levels. C) qRT-PCR analysis of Nedd4 RNA levels corrected for total GAPDH. D) Levels of Spy1 and Spy1-TST protein were assessed in HEK-293 cells after transfection with control vector pCS3, myc-Spy1-pCS3, and myc-Spy1-TST-pCS3 in the presence or absence of 50 J/m^2^ UV damage. Cells were collected 24 h after damage and subjected to Western blot analysis. Densitometry analysis was performed for total Spy1 protein levels and corrected for total Actin levels (n = 3). Error bars represent SE. *p < 0.05, **p < 0.01.

**Additional file 5: Figure S5.** A) qRT-PCR analysis of Spy1 levels in 8-week-old MMTV-Spy1 mice and their control littermates (cntl) 48 h after DMBA treatment in mice with and without DMBA. Levels of Flag-Spy1 are corrected for total levels of GAPDH. B) Representative western blot for p53 protein levels in MMTV-Spy1 8-week-old mice and their control littermates 48 h after DMBA treatment. Error bars represent SE. ***p < 0.001.

**Additional file 6: Figure S6.** A) Immunohistochemical analysis of Spy1 levels in inguinal glands of 8 week old wild-type and p53 heterozygous mice. Levels of Spy1 were quantified using ImageJ (left panel) and representative images are shown in the right panel. (Wild-type n = 3; p53 heterozygote *n* = 6) Scale bar = 100 μM. B) PCNA expression in MMTV-Spy1 and p53 heterozygous cross mice at 8 weeks of age via immunohistochemical analysis. Quantification of percentage of PCNA positive mammary epithelial cells over 5 fields of view per sample in left panel (WT n = 3; MMTV-Spy1 *n* = 4; p53+/− n = 6; MMTV-Spy1 p53+/− *n* = 5). Representative images shown in the right panel. Scale bar = 100 μM. Error bars represent SE. *p < 0.05, **p < 0.01, ***p < 0.001.

## Data Availability

All data generated from this study are included in the manuscript and Additional file 1 supplemental files.

## References

[1] Bartek J, Lukas J (2001). Pathways governing G1/S transition and their response to DNA damage. FEBS Lett.

[2] Meek DW (2004). The p53 response to DNA damage. DNA Repair (Amst).

[3] Sakaguchi K, Herrera JE, Saito S, Miki T, Bustin M, Vassilev A, Anderson CW, Appella E (1998). DNA damage activates p53 through a phosphorylation-acetylation cascade. Genes Dev.

[4] Sancar A, Lindsey-Boltz LA, Unsal-Kacmaz K, Linn S (2004). Molecular mechanisms of mammalian DNA repair and the DNA damage checkpoints. Annu Rev Biochem.

[5] Akashi M, Koeffler HP (1998). Li-Fraumeni syndrome and the role of the p53 tumor suppressor gene in cancer susceptibility. Clin Obstet Gynecol.

[6] Soussi T, Ishioka C, Claustres M, Beroud C (2006). Locus-specific mutation databases: pitfalls and good practice based on the p53 experience. Nat Rev Cancer.

[7] Hutchinson JN, Muller WJ (2000). Transgenic mouse models of human breast cancer. Oncogene.

[8] Donehower LA, Harvey M, Slagle BL, McArthur MJ, Montgomery CA, Butel JS, Bradley A (1992). Mice deficient for p53 are developmentally normal but susceptible to spontaneous tumours. Nature.

[9] Purdie CA, Harrison DJ, Peter A, Dobbie L, White S, Howie SE, Salter DM, Bird CC, Wyllie AH, Hooper ML (1994). Tumour incidence, spectrum and ploidy in mice with a large deletion in the p53 gene. Oncogene.

[10] Jacks T, Remington L, Williams BO, Schmitt EM, Halachmi S, Bronson RT, Weinberg RA (1994). Tumor spectrum analysis in p53-mutant mice. Curr Biol.

[11] Stratton MR, Rahman N (2008). The emerging landscape of breast cancer susceptibility. Nat Genet.

[12] Lenormand JL, Dellinger RW, Knudsen KE, Subramani S, Donoghue DJ (1999). Speedy: a novel cell cycle regulator of the G2/M transition. EMBO J.

[13] McAndrew CW, Gastwirt RF, Donoghue DJ (2009). The atypical CDK activator Spy1 regulates the intrinsic DNA damage response and is dependent upon p53 to inhibit apoptosis. Cell Cycle.

[14] Porter LA, Dellinger RW, Tynan JA, Barnes EA, Kong M, Lenormand JL, Donoghue DJ (2002). Human Speedy: a novel cell cycle regulator that enhances proliferation through activation of Cdk2. J Cell Biol.

[15] Barnes EA, Porter LA, Lenormand JL, Dellinger RW, Donoghue DJ (2003). Human Spy1 promotes survival of mammalian cells following DNA damage. Cancer Res.

[16] Gastwirt RF, Slavin DA, McAndrew CW, Donoghue DJ (2006). Spy1 expression prevents normal cellular responses to DNA damage: inhibition of apoptosis and checkpoint activation. J Biol Chem.

[17] Cheng A, Gerry S, Kaldis P, Solomon MJ (2005). Biochemical characterization of Cdk2-Speedy/Ringo A2. BMC Biochem.

[18] Karaiskou A, Perez LH, Ferby I, Ozon R, Jessus C, Nebreda AR (2001). Differential regulation of Cdc2 and Cdk2 by RINGO and cyclins. J Biol Chem.

[19] McGrath DA, Fifield BA, Marceau AH, Tripathi S, Porter LA, Rubin SM (2017). Structural basis of divergent cyclin-dependent kinase activation by Spy1/RINGO proteins. EMBO J.

[20] Golipour A, Myers D, Seagroves T, Murphy D, Evan GI, Donoghue DJ, Moorehead RA, Porter LA (2008). The Spy1/RINGO family represents a novel mechanism regulating mammary growth and tumorigenesis. Cancer Res.

[21] Zucchi I, Mento E, Kuznetsov VA, Scotti M, Valsecchi V, Simionati B, Vicinanza E, Valle G, Pilotti S, Reinbold R (2004). Gene expression profiles of epithelial cells microscopically isolated from a breast-invasive ductal carcinoma and a nodal metastasis. Proc Natl Acad Sci U S A.

[22] Al Sorkhy M, Ferraiuolo RM, Jalili E, Malysa A, Fratiloiu AR, Sloane BF, Porter LA (2012). The cyclin-like protein Spy1/RINGO promotes mammary transformation and is elevated in human breast cancer. BMC Cancer.

[23] Ke Q, Ji J, Cheng C, Zhang Y, Lu M, Wang Y, Zhang L, Li P, Cui X, Chen L (2009). Expression and prognostic role of Spy1 as a novel cell cycle protein in hepatocellular carcinoma. Exp Mol Pathol.

[24] Lubanska D, Market-Velker BA, de Carvalho AC, Mikkelsen T, Fidalgo da Silva E, Porter LA (2014). The cyclin-like protein Spy1 regulates growth and division characteristics of the CD133+ population in human glioma. Cancer Cell.

[25] Hang Q, Fei M, Hou S, Ni Q, Lu C, Zhang G, Gong P, Guan C, Huang X, He S (2012). Expression of Spy1 protein in human non-Hodgkin’s lymphomas is correlated with phosphorylation of p27 Kip1 on Thr187 and cell proliferation. Med Oncol.

[26] Porter LA, Kong-Beltran M, Donoghue DJ (2003). Spy1 interacts with p27Kip1 to allow G1/S progression. Mol Biol Cell.

[27] Mroue R, Bissell MJ (2013). Three-dimensional cultures of mouse mammary epithelial cells. Methods Mol Biol.

[28] Al Sorkhy M, Craig R, Market B, Ard R, Porter LA (2009). The cyclin-dependent kinase activator, Spy1A, is targeted for degradation by the ubiquitin ligase NEDD4. J Biol Chem.

[29] Boyd SD, Tsai KY, Jacks T (2000). An intact HDM2 RING-finger domain is required for nuclear exclusion of p53. Nat Cell Biol.

[30] Shin HJ, Kim H, Oh S, Lee JG, Kee M, Ko HJ, Kweon MN, Won KJ, Baek SH (2016). AMPK-SKP2-CARM1 signalling cascade in transcriptional regulation of autophagy. Nature.

[31] Vargo-Gogola T, Heckman BM, Gunther EJ, Chodosh LA, Rosen JM (2006). P190-B Rho GTPase-activating protein overexpression disrupts ductal morphogenesis and induces hyperplastic lesions in the developing mammary gland. Mol Endocrinol.

[32] Sleeman KE, Kendrick H, Ashworth A, Isacke CM, Smalley MJ (2006). CD24 staining of mouse mammary gland cells defines luminal epithelial, myoepithelial/basal and non-epithelial cells. Breast Cancer Res.

[33] Lee HJ, Lee YJ, Kang CM, Bae S, Jeoung D, Jang JJ, Lee SS, Cho CK, Lee YS (2008). Differential gene signatures in rat mammary tumors induced by DMBA and those induced by fractionated gamma radiation. Radiat Res.

[34] Hoshino A, Yee CJ, Campbell M, Woltjer RL, Townsend RL, van der Meer R, Shyr Y, Holt JT, Moses HL, Jensen RA (2007). Effects of BRCA1 transgene expression on murine mammary gland development and mutagen-induced mammary neoplasia. Int J Biol Sci.

[35] Das SK, Delp CR, Bandyopadhyay AM, Mathiesen M, Baird WM, Banerjee MR (1989). Fate of 7,12-dimethylbenz(a) anthracene in the mouse mammary gland during initiation and promotion stages of carcinogenesis in vitro. Cancer Res.

[36] Merlo GR, Venesio T, Taverna D, Callahan R, Hynes NE (1993). Growth suppression of normal mammary epithelial cells by wild-type p53. Ann N Y Acad Sci.

[37] Ogretmen B, Safa AR (1997). Expression of the mutated p53 tumor suppressor protein and its molecular and biochemical characterization in multidrug resistant MCF-7/Adr human breast cancer cells. Oncogene.

[38] Kato S, Han SY, Liu W, Otsuka K, Shibata H, Kanamaru R, Ishioka C (2003). Understanding the function-structure and function-mutation relationships of p53 tumor suppressor protein by high-resolution missense mutation analysis. Proc Natl Acad Sci U S A.

[39] Dinarina A, Santamaria PG, Nebreda AR (2009). Cell cycle regulation of the mammalian CDK activator RINGO/Speedy A. FEBS Lett.

[40] Ullrich RL, Bowles ND, Satterfield LC, Davis CM (1996). Strain-dependent susceptibility to radiation-induced mammary cancer is a result of differences in epithelial cell sensitivity to transformation. Radiat Res.

[41] Wang XD, Zhu MW, Shan D, Wang SY, Yin X, Yang YQ, Wang TH, Zhang CT, Wang Y, Liang WW (2019). Spy1, a unique cell cycle regulator, alters viability in ALS motor neurons and cell lines in response to mutant SOD1-induced DNA damage. DNA Repair (Amst).

[42] Currier N, Solomon SE, Demicco EG, Chang DL, Farago M, Ying H, Dominguez I, Sonenshein GE, Cardiff RD, Xiao ZX (2005). Oncogenic signaling pathways activated in DMBA-induced mouse mammary tumors. Toxicol Pathol.

[43] Rehm S (1990). Chemically induced mammary gland adenomyoepitheliomas and myoepithelial carcinomas of mice. Immunohistochemical and ultrastructural features. Am J Pathol.

[44] Kuperwasser C, Hurlbut GD, Kittrell FS, Dickinson ES, Laucirica R, Medina D, Naber SP, Jerry DJ (2000). Development of spontaneous mammary tumors in BALB/c p53 heterozygous mice. A model for Li-Fraumeni syndrome. Am J Pathol.

[45] Ponnaiya B, Cornforth MN, Ullrich RL (1997). Radiation-induced chromosomal instability in BALB/c and C57BL/6 mice: the difference is as clear as black and white. Radiat Res.

[46] Miki Y, Swensen J, Shattuck-Eidens D, Futreal PA, Harshman K, Tavtigian S, Liu Q, Cochran C, Bennett LM, Ding W (1994). A strong candidate for the breast and ovarian cancer susceptibility gene BRCA1. Science.

[47] Wooster R, Bignell G, Lancaster J, Swift S, Seal S, Mangion J, Collins N, Gregory S, Gumbs C, Micklem G (1995). Identification of the breast cancer susceptibility gene BRCA2. Nature.

[48] Couch FJ, Kuchenbaecker KB, Michailidou K, Mendoza-Fandino GA, Nord S, Lilyquist J, Olswold C, Hallberg E, Agata S, Ahsan H (2016). Identification of four novel susceptibility loci for oestrogen receptor negative breast cancer. Nat Commun.

[49] Arason A, Gunnarsson H, Johannesdottir G, Jonasson K, Bendahl PO, Gillanders EM, Agnarsson BA, Jonsson G, Pylkas K, Mustonen A (2010). Genome-wide search for breast cancer linkage in large Icelandic non-BRCA1/2 families. Breast Cancer Res.

[50] Smith P, McGuffog L, Easton DF, Mann GJ, Pupo GM, Newman B, Chenevix-Trench G, Szabo C, Southey M, Renard H (2006). A genome wide linkage search for breast cancer susceptibility genes. Genes Chromosomes Cancer.

