# Supplementary figures and images for: The atypical cyclin-like protein Spy1 overrides p53-mediated tumour suppression and promotes susceptibility to breast tumourigenesis
Source: Breast Cancer Res. 2019 Dec 11;21:140. doi: 10.1186/s13058-019-1211-3 (PMC6907270; doi:10.1186/s13058-019-1211-3)

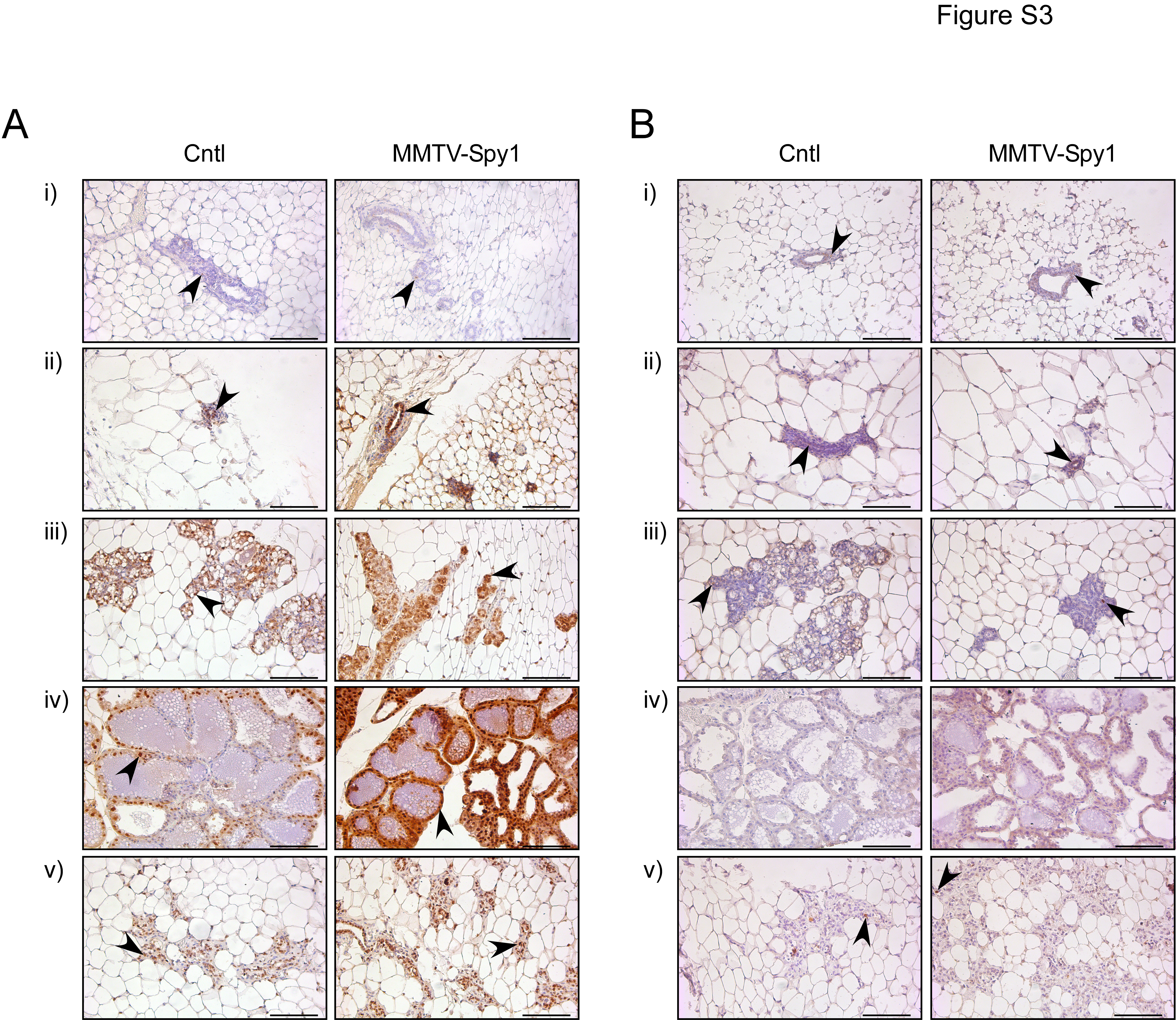

Supplement: Supplementary file 3 — Additional file 3: Figure S3. Related to Figure 1: Spy1 increases proliferation during development in mammary epithelial cells. Representative images are shown of A) PCNA and B) cleaved caspase 3 at i) 8 week puberty, ii) 12 week adult, iii) 16.5 day pregnancy, iv) 4 day lactation and v) 4 day involution. Arrowheads point to A) PCNA and B) cleaved caspase 3 positive cells. Scale bar= 100 μm. [file 13058_2019_1211_MOESM3_ESM.tif]

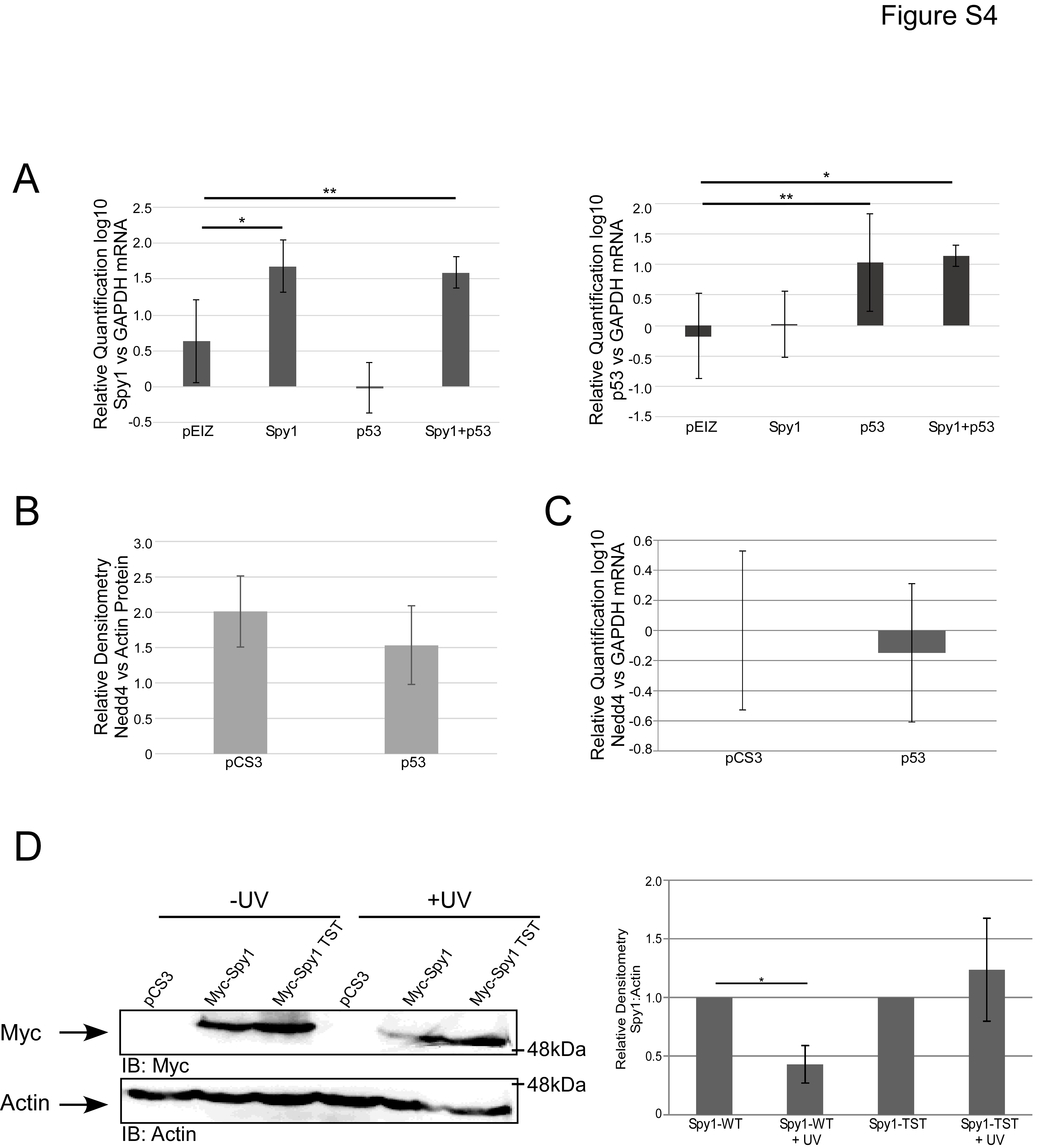

Supplement: Supplementary file 4 — Additional file 4: Figure S4. A) Spy1 and p53 were overexpressed in MDA-MB-231 cells to determine if p53 can alter Spy1 mRNA levels (n = 3). B & C) p53 or control vector was overexpressed in HEK-293 cells to assess protein and RNA levels of Nedd4. B) Western blot analysis of Nedd4 protein levels corrected for total Actin protein levels. C) qRT-PCR analysis of Nedd4 RNA levels corrected for total GAPDH. D) Levels of Spy1 and Spy1-TST protein were assessed in HEK-293 cells after transfection with control vector pCS3, myc-Spy1-pCS3, and myc-Spy1-TST-pCS3 in the presence or absence of 50 J/m2 UV damage. Cells were collected 24 h after damage and subjected to Western blot analysis. Densitometry analysis was performed for total Spy1 protein levels and corrected for total Actin levels (n = 3). Error bars represent SE. *p < 0.05, **p < 0.01. [file 13058_2019_1211_MOESM4_ESM.tif]

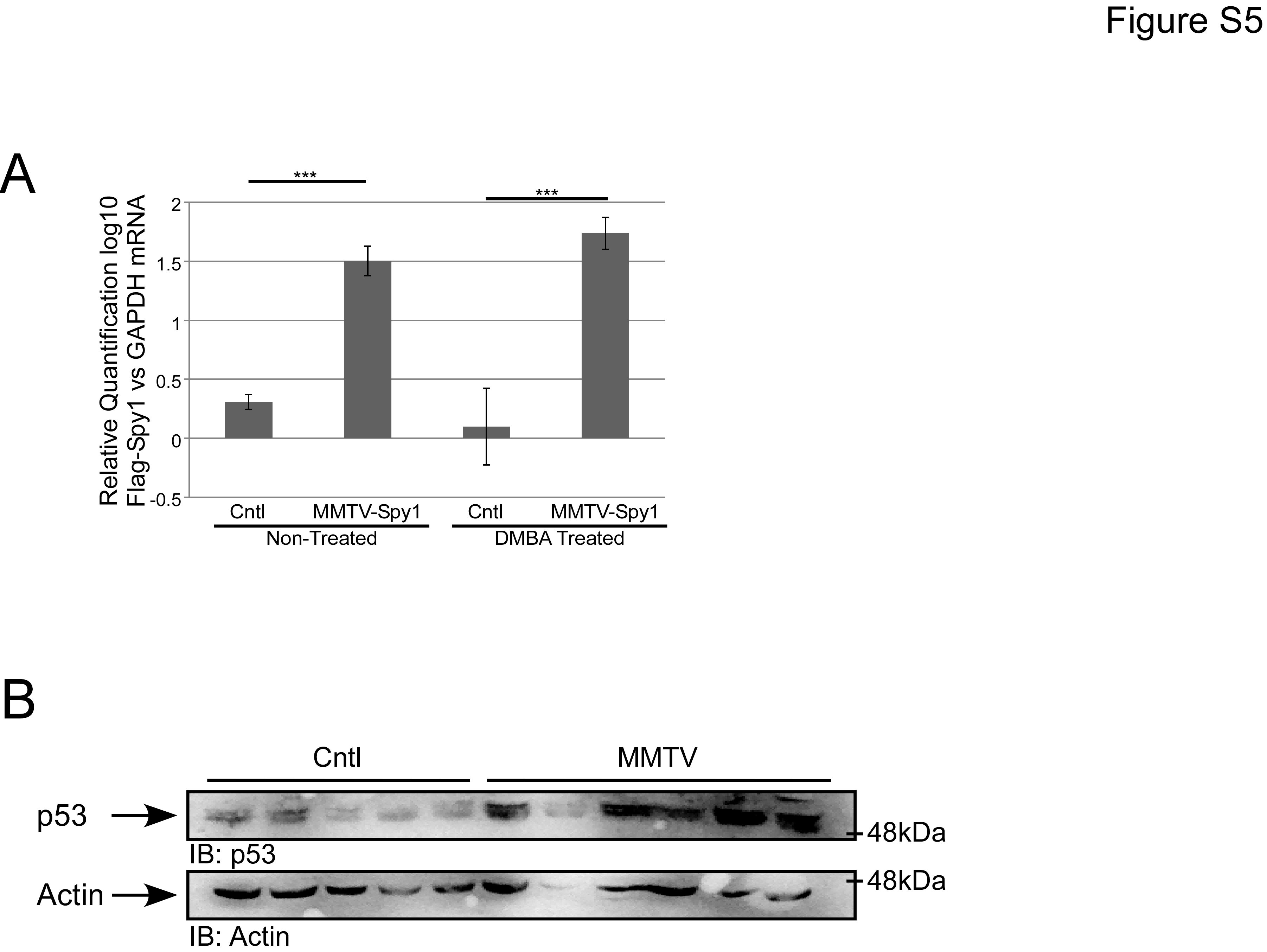

Supplement: Supplementary file 5 — Additional file 5: Figure S5. A) qRT-PCR analysis of Spy1 levels in 8-week-old MMTV-Spy1 mice and their control littermates (cntl) 48 h after DMBA treatment in mice with and without DMBA. Levels of Flag-Spy1 are corrected for total levels of GAPDH. B) Representative western blot for p53 protein levels in MMTV-Spy1 8-week-old mice and their control littermates 48 h after DMBA treatment. Error bars represent SE. ***p < 0.001. [file 13058_2019_1211_MOESM5_ESM.tif]

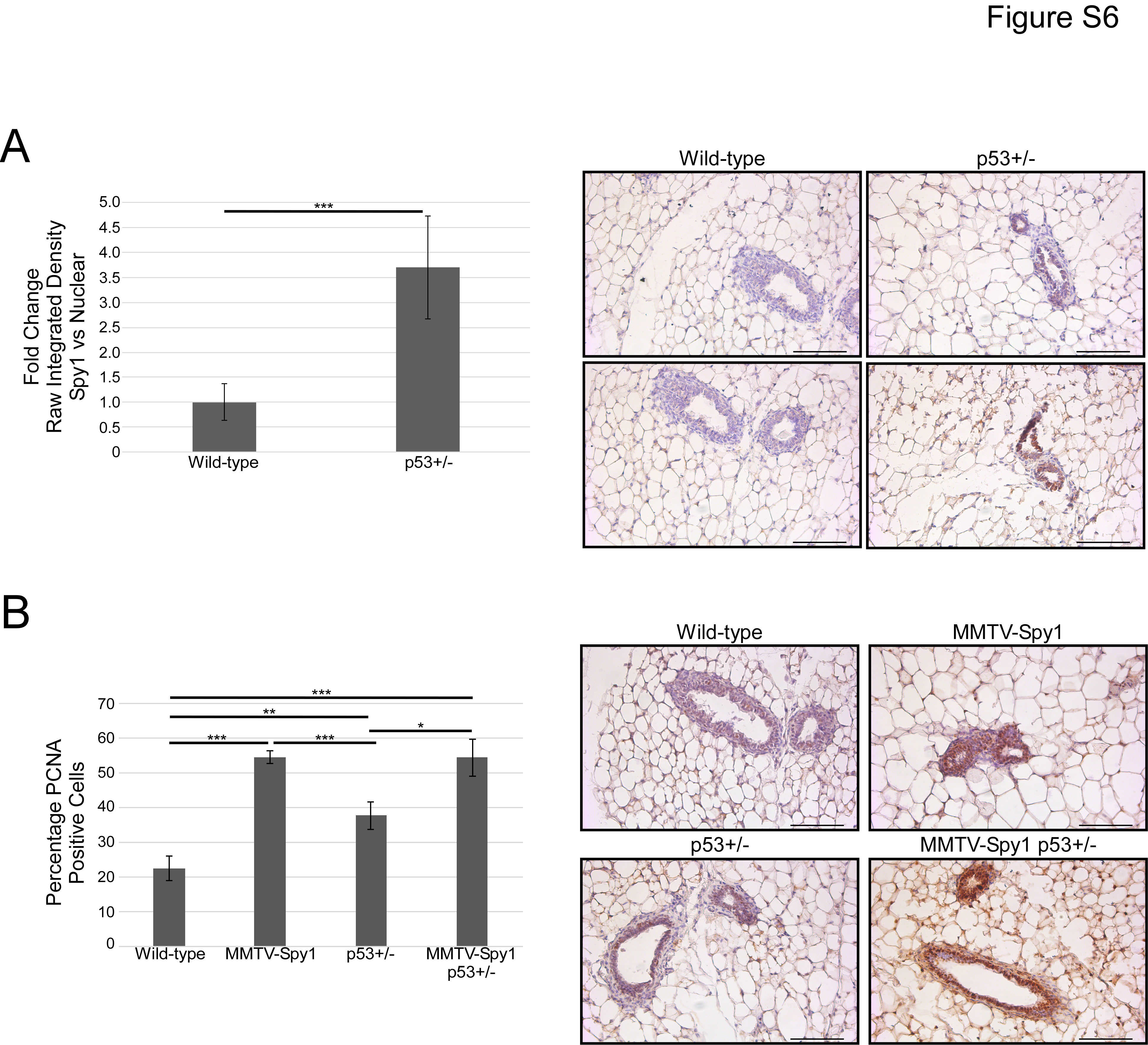

Supplement: Supplementary file 6 — Additional file 6: Figure S6. A) Immunohistochemical analysis of Spy1 levels in inguinal glands of 8 week old wild-type and p53 heterozygous mice. Levels of Spy1 were quantified using ImageJ (left panel) and representative images are shown in the right panel. (Wild-type n = 3; p53 heterozygote n = 6) Scale bar = 100 μM. B) PCNA expression in MMTV-Spy1 and p53 heterozygous cross mice at 8 weeks of age via immunohistochemical analysis. Quantification of percentage of PCNA positive mammary epithelial cells over 5 fields of view per sample in left panel (WT n = 3; MMTV-Spy1 n = 4; p53+/− n = 6; MMTV-Spy1 p53+/− n = 5). Representative images shown in the right panel. Scale bar = 100 μM. Error bars represent SE. *p < 0.05, **p < 0.01, ***p < 0.001. [file 13058_2019_1211_MOESM6_ESM.tif]
